# Clinical spectrum and genotype–phenotype correlations of *MT-CYB*-associated mitochondrial disease: from the m.15045G > A variant to the broader disease landscape

**DOI:** 10.3389/fnins.2026.1968308

**Published:** 2026-09-29

**Authors:** Zimeng He, Tongyue Li, Chaolong Xu, Yang Liu, Ying Zou, Zhimei Liu, Jiatong Xu, Xuejing Kang, Huafang Jiang, Danmin Shen, Yunxi Zhang, Hui Xiong, Fang Fang

**Affiliations:** 1 Department of Neurology, Beijing Children's Hospital, National Center for Children's Health, Capital Medical University, Beijing, China; 2 Department of Pathology, Beijing Children's Hospital, National Center for Children's Health, Capital Medical University, Beijing, China; 3 Department of Pediatrics, Weifang Maternal and Child Health Hospital, Weifang, Shandong, China

**Keywords:** complex III (cytochrome bc1 complex), genotype–phenotype correlation, m.15045G > A, mitochondrial disease, mitochondrial myopathy, *MT-CYB* gene

## Abstract

**Aim:**

To characterize the clinical and molecular features associated with the *MT-CYB* m.15045G > A variant and investigate the clinical spectrum and genotype–phenotype relationships of *MT-CYB*-associated mitochondrial disease.

**Methods:**

We performed comprehensive clinical, metabolic, histopathological, genetic, and mitochondrial functional analyses in a patient carrying the m.15045G > A variant. A systematic literature review of previously reported disease-associated *MT-CYB* variants was conducted, followed by genotype–phenotype comparisons and unsupervised hierarchical clustering integrating clinical, genetic, and functional characteristics.

**Results:**

The patient presented with exercise intolerance, borderline intellectual functioning, and elevated medium and long-chain acylcarnitines. Muscle biopsy showed mitochondrial myopathic changes, and respiratory chain analysis demonstrated isolated complex III (CIII) deficiency. The m.15045G > A variant showed tissue-specific heteroplasmy of 22.5, 70.5, and 95.5% in blood, urine, and muscle, respectively, providing further evidence for its pathogenicity. Among 34 patients with disease-associated *MT-CYB* variants, mitochondrial myopathy (MM) was the most common phenotype (50.0%). Patients with MM showed a later age at onset, and muscle-restricted variants were markedly enriched in the MM group (*p* < 0.001). In contrast, muscle heteroplasmy level, variant type, and isolated CIII deficiency were not significantly associated with phenotype. Hierarchical clustering showed 88.2% concordance with clinical phenotype classification.

**Conclusion:**

Our findings provide further evidence for the pathogenicity of m.15045G > A and broaden its associated clinical and metabolic features. Tissue-specific distribution of *MT-CYB* variants showed an association with clinical phenotype, while other genetic and functional factors may collectively contribute to phenotypic variability.

## Introduction

Complex III (CIII) is composed of 11 subunits, among which cytochrome b, encoded by *MT-CYB*, is the only subunit encoded by the mitochondrial genome (Bénit et al., 2009). Variants in *MT-CYB* cause isolated CIII deficiency, a rare oxidative phosphorylation (OXPHOS) disorder with considerable clinical heterogeneity, manifesting as exercise intolerance, cardiomyopathy, Leber hereditary optic neuropathy (LHON), and Leigh syndrome (LS) (Hagen et al., 2013; Petrovic Pajic et al., 2025; Tropeano et al., 2025; Andreu et al., 1999). Pathogenic *MT-CYB* variants encompass diverse variant types, including missense, nonsense, frameshift, and in-frame deletions (Andreu et al., 1999; Mori et al., 2015; Carossa et al., 2014). Despite this broad clinical spectrum, the determinants of phenotypic variability in *MT-CYB*-associated disease remain poorly understood. In particular, the contributions of variant characteristics, heteroplasmy, tissue distribution of *MT-CYB* variants, and biochemical abnormalities to clinical phenotype have not been systematically evaluated. The rarity of pathogenic *MT-CYB* variants and the limited number of well-characterized patients further hinder the establishment of clear genotype–phenotype relationships.

The *MT-CYB* m.15045G > A variant was previously reported in an adult patient with isolated mitochondrial myopathy characterized by progressive exercise intolerance and elevated medium and long-chain acylcarnitines, but its pathogenicity remains insufficiently characterized (Kaphan et al., 2018). Here, we report the second patient carrying this variant, presenting with childhood-onset exercise intolerance, borderline intellectual functioning, and a similar acylcarnitine profile, and provide further biochemical and functional evidence for its pathogenicity. In addition, we systematically reviewed previously reported *MT-CYB* variants to characterize the clinical and variant spectrum of *MT-CYB*-associated mitochondrial disease and performed genotype–phenotype analyses to explore potential factors associated with phenotypic variability.

## Materials and methods

### Patient and clinical data

A pediatric patient with suspected mitochondrial disease was included in this study. The patient underwent comprehensive clinical evaluation and laboratory investigations as part of the diagnostic workup. Clinical data were collected from medical records, including demographic characteristics, age at onset, initial symptoms, clinical manifestations, laboratory findings, neuroimaging features, treatment, and clinical outcomes. This study was approved by the Ethics Committee of Beijing Children’s Hospital, and written informed consent was obtained from the patient’s guardians.

### Muscle biopsy and histologic analysis

Muscle specimens were obtained by open biopsy, snap-frozen, and stored in liquid nitrogen. The samples were evaluated by light microscopy after hematoxylin and eosin (H&E), modified Gomori trichrome (MGT), succinate dehydrogenase (SDH), cytochrome c oxidase (COX), NADH-tetrazolium reductase (NADH-TR), periodic acid–Schiff (PAS), and Oil Red O (ORO) staining. Transmission electron microscopy (TEM) was performed to assess mitochondrial morphology and ultrastructural abnormalities.

### Metabolic profiling

Amino acid and acylcarnitine profiles in dried blood spot samples were analyzed using ultra-performance liquid chromatography–tandem mass spectrometry (UPLC-MS/MS), and urinary organic acid profiles were analyzed using gas chromatography–mass spectrometry (GC–MS).

### Mitochondrial functional assessment

Mitochondrial respiratory chain (MRC) complex I, II, III, IV, combined complex II + III, and the mitochondrial marker enzyme citrate synthase (CS) activities were measured in the crude supernatant of muscle homogenates after centrifugation at 600 g, as described previously (Ogawa et al., 2017). Enzyme activities were normalized to appropriate reference enzymes, including CS or complex II, and expressed as percentages of the normal control mean. The laboratory reference values were based on the established MRC assay system described previously, with the normal control mean derived from skeletal muscle samples from 35 healthy adult controls (Higuchi et al., 2018). Long-term laboratory control/reference materials were repeatedly assessed for routine quality control and monitoring of the established assay reference system (Supplementary Table S1). In addition, a skeletal muscle sample (biceps brachii) from a healthy 15-year-old female was assayed concurrently with the patient sample as an internal assay control. MRC complex activities normalized to CS or complex II < 30% of the normal control mean were considered decreased according to established biochemical diagnostic criteria (Bernier et al., 2002). Skin fibroblasts were obtained from skin biopsies and cultured in Dulbecco’s Modified Eagle Medium (DMEM, Thermo Fisher Scientific) supplemented with 1% penicillin/streptomycin and 10% fetal bovine serum. Skin fibroblasts from the patient and one sex-matched healthy control (female, 9 years of age; upper-arm dermis; passage 4) were used for oxygen consumption rate (OCR) measurement using a XF96 Extracellular Flux Analyzer (Seahorse Bioscience, Billerica, MA, United States), as described previously (Ogawa et al., 2017). The control fibroblast cell line was maintained in our laboratory and had been previously characterized together with an independent healthy control fibroblast line, showing a comparable mitochondrial respiratory profile (Jiang et al., 2025). Fibroblasts were seeded at a density of 1.5–2.0 × 10^4^ cells per well in a 96-well plate. Five independent experiments were performed, each with triplicate technical wells, and the results were expressed as mean ± SD. Maximal respiration rate (MRR) was calculated as the FCCP-stimulated maximal OCR minus the rotenone-insensitive OCR and was expressed as a percentage of the control value. Based on the reference established by Ogawa et al. (2017), an MRR < 71.6% of the control level was considered significantly reduced.

### Genetic analysis

Genomic DNA was extracted from peripheral blood samples of the patient and her parents, and mitochondrial DNA (mtDNA) was extracted from peripheral blood, urine, and muscle tissue. To identify causative variants, whole-exome sequencing (WES) and whole-genome sequencing (WGS) were performed on peripheral blood-derived DNA using an Illumina sequencing platform, and complete mitochondrial genome sequencing was performed on multiple tissue samples using long-range PCR amplification followed by next-generation sequencing. Variant pathogenicity was determined according to standards and guidelines for the interpretation of sequence variants published in 2015 and 2020 (Richards et al., 2015; McCormick et al., 2020). Sanger sequencing was performed to validate the identified variants. The candidate mtDNA variant was also assessed in cultured skin fibroblasts, and the heteroplasmy level was estimated.

### RNA sequencing analysis

Total RNA extracted from patient-derived fibroblasts was subjected to transcriptome sequencing (RNA-seq) to evaluate aberrant gene expression and abnormal splicing events. Poly(A)-selected mRNA libraries were prepared and sequenced using a paired-end strategy. Sequence reads were aligned to the human reference genome (GRCh38/hg38), and aberrant expression and splicing analyses were performed using the standard analytical pipeline of the testing laboratory.

### Protein structural modeling and conservation analysis

Multiple sequence alignment of MT-CYB orthologs from different species was performed using Jalview software to evaluate the evolutionary conservation of the arginine residue at position 100 (R100). The amino acid sequence of human MT-CYB was retrieved from the NCBI database, and three-dimensional structural models of the wild-type and mutant proteins were generated using the SWISS-MODEL platform. The local conformation of residue 100 was visualized and analyzed using PyMOL software, with a focus on comparing hydrogen bond interactions before and after the variant.

### Literature review

A systematic literature review was conducted following the Preferred Reporting Items for Systematic Reviews and Meta-Analyses (PRISMA) guidelines (Stewart et al., 2015). A comprehensive search was performed in PubMed and EMBASE databases without language restrictions. The detailed search strategies are provided in Supplementary Table S2. The reference lists of all eligible articles were also manually screened to identify additional relevant studies. Studies were included if they met the following criteria: (1) reported one or more patients carrying a genetically confirmed *MT-CYB* variant with evidence supporting a potential disease-causing role; (2) provided individual-level clinical information, including at least demographic characteristics or clinical manifestations. Studies reporting aggregated data were included only when individual patient data could be extracted. Cases were excluded if: (1) the *MT-CYB* variant was not confirmed by genetic testing; (2) the original report provided insufficient evidence to support a potential disease association of the variant. Studies excluded for the latter reason are listed in Supplementary Table S3. Because variant interpretation frameworks have evolved substantially over the period covered by the literature review, inclusion was based on the evidence available in the original reports rather than restricted to variants currently classified as pathogenic or likely pathogenic. Specifically, only variants for which the original reports provided evidence supporting a potential disease-causing role, based on clinical, genetic, biochemical, histopathological, and/or functional findings, were considered eligible. All included *MT-CYB* variants were subsequently reassessed against contemporary ClinVar classifications and the ClinGen Mitochondrial Disease Nuclear and Mitochondrial Variant Curation Expert Panel (VCEP) recommendations (accessed September 3, 2026). Contemporary database classifications were not applied retrospectively as exclusion criteria, as several historically reported disease-associated *MT-CYB* variants with supportive evidence are currently classified as VUS under mtDNA-specific ACMG/AMP criteria. Current ClinVar and ClinGen classifications of all included variants are provided in Supplementary Table S4. For patients reported in multiple publications, data from all relevant reports were reviewed and integrated into a single case to avoid duplicate counting.

Data extracted from each eligible case included demographic information, genotype, heteroplasmy and inheritance, clinical manifestations, biochemical and neuroimaging findings, muscle pathology, respiratory chain enzyme activities, and outcome. For phenotypic classification, non-syndromic mitochondrial disease (NSMD) was used as a study-defined operational category for patients with mitochondrial disease who did not fit the other predefined clinical phenotype categories. Variables that could not be determined from the original reports were recorded as “unknown” and were excluded from the corresponding analyses. A total of 29 articles comprising 33 individual cases were finally included (Petrovic Pajic et al., 2025; Tropeano et al., 2025; Andreu et al., 1999; Mori et al., 2015; Carossa et al., 2014; Kaphan et al., 2018; Dumoulin et al., 1996; Mousson et al., 1995; Andreu et al., 1998; Andreu et al., 1999; Valnot et al., 1999; De Coo et al., 1999; Andreu et al., 2000; Papadimitriou et al., 1984; Keightley et al., 2000; Wibrand et al., 2001; Legros et al., 2001; Schuelke et al., 2002; Lamantea et al., 2002; Mancuso et al., 2003; Bruno et al., 2003; Blakely et al., 2005; Fragaki et al., 2009; Massie et al., 2010; Emmanuele et al., 2013; Mancuso et al., 2014; Chaussenot et al., 2018; Barcia et al., 2020; Zarrouk et al., 2021), and the study selection process is illustrated in the PRISMA flow diagram (Figure 1).

**Figure 1:**
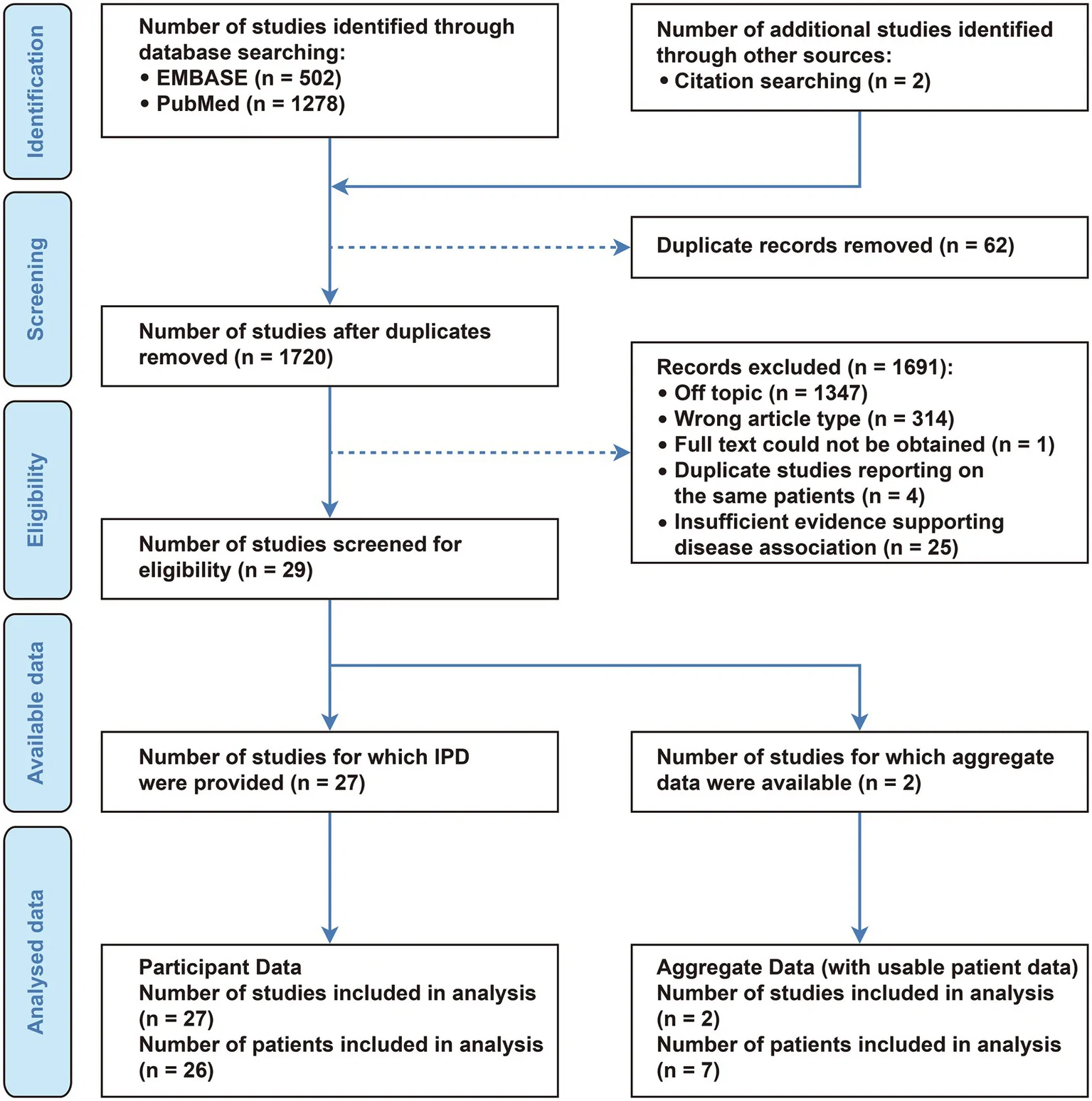
Flow diagram for literature review following the PRISMA-IPD (preferred reporting items for systematic reviews and meta-analyses of individual participant data) guidelines (Stewart et al., 2015).

### Genotype–phenotype correlation analysis

Cases with available clinical and genetic data were included in the genotype–phenotype correlation analysis. Based on the predominant clinical phenotype, patients were classified into mitochondrial myopathy (MM) and non-MM groups. Demographic, genetic, and functional characteristics were compared between the two groups to explore potential genotype–phenotype associations. Cases with missing data for a given variable were excluded from the corresponding analysis. Furthermore, unsupervised hierarchical clustering was performed using six selected genetic and functional features, and the resulting clusters were compared with the clinical phenotype classification (MM versus non-MM) to assess the concordance between feature-based clustering and clinical phenotypes.

### Statistical analysis

Data normality was assessed using the Shapiro–Wilk test. Continuous variables are presented as median [P25, P75] and were compared using the Mann–Whitney U test. Categorical variables are presented as counts and percentages and were compared using the chi-square (*χ*^2^) test or Fisher’s exact test, as appropriate. Unsupervised hierarchical clustering was performed using Gower distance with average linkage. A two-sided *p* value < 0.05 was considered statistically significant. Statistical analyses were performed using R (version 4.4.2), IBM SPSS Statistics (version 27.0.1), and GraphPad Prism (version 10.1.2).

## Results

### Clinical features

The proband was a 12-year-6-month-old girl born at full term by spontaneous vaginal delivery. She was the first child of non-consanguineous parents. Her neonatal course was uneventful, and her early growth and developmental milestones were reported to be normal. At 8 years of age, she was noted to have poor weight gain, prolonged mealtimes, occasional nausea, and vomiting. Reduced exercise endurance compared with her peers was also observed. She attended mainstream school; however, her academic performance was poor, with difficulties in attention, comprehension, and learning. Her exercise intolerance and fatigability gradually worsened over the following 5 years. At presentation, her body weight was 33 kg (3rd–10th percentiles for age and sex), and her height was 148 cm (10th–25th percentiles for age and sex). Neurological examination revealed normal gait, negative Gower’s sign, and preserved ability to jump on both or single feet and walk on heels or toes. Muscle strength and muscle tone were normal.

Biochemical testing showed hyperlactatemia, with blood lactate levels ranging from 4.79 to 6.42 mmol/L (reference range: 0.5–2.2 mmol/L). Creatine kinase, cardiac enzymes, and liver and renal function tests were within normal limits. Brain MRI revealed prominence of the cerebral and cerebellar sulci and a mega cisterna magna, with no other abnormalities identified (Figures 2A,a,b). Thigh muscle MRI demonstrated mildly increased signal intensity on T2 fat-suppressed images, suggestive of mild muscle edema (Figures 2A,c). The six-minute walk test showed a walking distance of 360 m, accompanied by subjective fatigue and a Borg dyspnea scale score of 1. Wechsler intelligence testing revealed a full-scale intelligence quotient (FSIQ) of 79, consistent with borderline intellectual functioning. Electroencephalography and electromyography were unremarkable.

**Figure 2:**
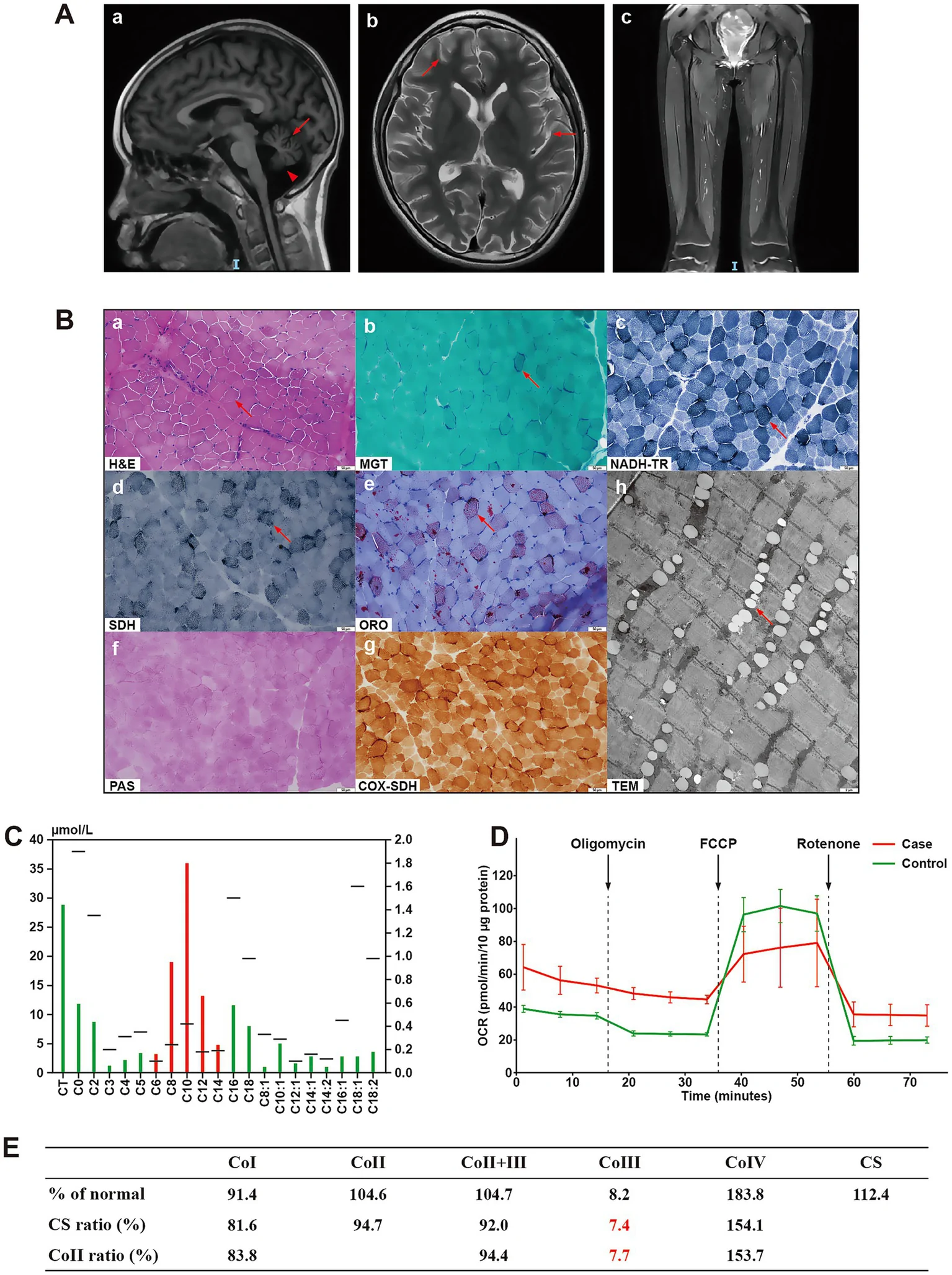
Clinical, pathological, metabolic, and functional characterization of the proband. **(A)** Brain and muscle MRI at 12 years of age. **(a,b)** Brain MRI showing prominence of the cerebral and cerebellar sulci (arrows) and a mega cisterna magna (arrowhead). **(c)** Thigh muscle MRI demonstrating mild muscle edema. **(B)** Muscle pathology. **(a)** H&E staining showing variation in muscle fiber diameter and basophilic granular changes. **(b)** MGT staining showing ragged-red fibers (RRFs). **(c)** NADH-TR staining showing intense staining of RRFs. **(d)** SDH staining showing ragged-blue fibers (RBFs). **(e)** ORO staining showing lipid droplet accumulation. **(f)** PAS staining showing no abnormal glycogen accumulation. **(g)** COX-SDH staining showing preserved COX activity without COX-deficient fibers. **(h)** TEM showing mitochondrial accumulation and increased lipid droplets. Scale bars = 2 μm. **(C)** Acylcarnitine profile. Blood acylcarnitine profile showing elevated medium and long-chain acylcarnitines. C0, C2 and C3 are to be read on left scale while all other acylcarnitines are to be read on right scale. Black horizontal lines indicate the upper limits of the reference ranges, and elevated acylcarnitine species are highlighted in red. **(D)** Oxygen consumption rate (OCR). OCR profiles of patient-derived fibroblasts and healthy controls following sequential treatment with oligomycin, FCCP, and rotenone. **(E)** Mitochondrial respiratory chain (MRC) enzyme activities. MRC enzyme activities in skeletal muscle expressed as percentages of the normal control level, CS-normalized activities, and CoII-normalized activities, demonstrating an isolated CIII deficiency. Enzyme activities of CoI, CoII, and CS are expressed as nmol·min^−1^·mg protein^−1^; activities of CoIII and CoIV are expressed as first-order rate constants (nmol^−1^·min^−1^). H&E, hematoxylin and eosin; MGT, modified Gomori trichrome; NADH-TR, NADH-tetrazolium reductase; SDH, succinate dehydrogenase; ORO, Oil Red O; PAS, periodic acid–Schiff; COX, cytochrome c oxidase; TEM, transmission electron microscopy; OCR, oxygen consumption rate; FCCP, carbonyl cyanide phenylhydrazone.

Given the clinical suspicion of mitochondrial disease, the patient received supportive therapy with L-carnitine (1 g once daily), idebenone (60 mg twice daily), coenzyme Q10 (100 mg twice daily), vitamin B complex (100 mg twice daily), and creatine monohydrate (2 g twice daily), together with moderate exercise training. During the 6-month follow-up, her clinical condition remained stable, and she reported a slight subjective improvement in fatigability. The most recent blood lactate level was 4.15 mmol/L (reference range: 0.5–2.2 mmol/L). However, no objective assessment of exercise capacity was performed during follow-up. No treatment-related adverse effects were observed.

### Muscle biopsy findings

Muscle biopsy revealed mitochondrial myopathic changes. On H&E staining, mild variation in muscle fiber diameter, scattered atrophic fibers, and numerous basophilic granular changes were observed. MGT staining demonstrated numerous ragged-red fibers (RRFs). NADH-TR staining showed intense staining of RRFs, and SDH staining showed numerous ragged-blue fibers (RBFs). COX-SDH staining showed preserved COX activity, with no COX-deficient fibers identified. ORO staining demonstrated moderate lipid droplet accumulation in many myofibers, whereas PAS staining showed no abnormal glycogen accumulation. TEM revealed mild myofiber atrophy, increased numbers of mitochondria in the intermyofibrillar and subsarcolemmal regions with focal mitochondrial aggregates, and increased lipid droplets interspersed among mitochondria (Figure 2B).

### Metabolic findings

Blood acylcarnitine profiling demonstrated elevations of multiple medium and long-chain acylcarnitines, including C6, C8, C10, C12, and C14, together with increased acylcarnitine ratios (C8/C2, C8/C3, C8/C16, and C14:1/C16) (Figure 2C). Urinary organic acid analysis showed markedly elevated lactate, accompanied by mild elevations of 2-hydroxybutyrate, ketone bodies, glutarate, and 2-hydroxyglutarate.

### Mitochondrial functional assessment

OCR analysis in patient-derived fibroblasts demonstrated an OCR of 76.9% relative to healthy controls, which remained within the normal reference range but was close to the lower limit (Figure 2D). MRC enzyme analysis of skeletal muscle demonstrated an isolated CIII deficiency, with CIII activity reduced to approximately 8.2% of the normal reference level and to 7.4 and 7.7% after normalization to CS and complex II, respectively. In contrast, the activities of complexes I, II, and IV, as well as their corresponding ratios to CS and CII, were within the normal ranges (Figure 2E). A healthy muscle control was assayed concurrently, with the corresponding enzyme activities provided in Supplementary Table S5.

### Genetic findings

WES and WGS did not identify any pathogenic or likely pathogenic variants in nuclear genes associated with mitochondrial disease. RNA sequencing revealed no clinically relevant aberrant gene expression or alternative splicing (fibroblast RNA-seq generated 23.91 Gb of data comprising 159,429,076 reads, with a mapping rate of 99.85%, Q20 of 99.22%, and an rRNA proportion of 2.79%; detailed expression and splicing profiles of key nuclear-encoded CIII genes are provided in Supplementary Table S6). Full-length mtDNA sequencing did not identify other known pathogenic mtDNA variants but revealed a heteroplasmic *MT-CYB* variant, m.15045G > A (p. Arg100Gln), in the proband. The variant was detected in peripheral blood, urine, and skeletal muscle, with heteroplasmy levels of 22.5, 70.5, and 95.5%, respectively (Figure 3A). It was also detected in cultured skin fibroblasts, with an estimated heteroplasmy level of approximately 35%. Sanger sequencing confirmed the presence of the variant in the proband, and the variant was not detected in the mother’s blood or urine samples (Figure 3B). According to the 2020 ACMG/AMP specifications for mitochondrial DNA variants, the variant was classified as likely pathogenic (PS2_Moderate, PS4_Supporting, PM2_Supporting, PP3, and PP4).

**Figure 3:**
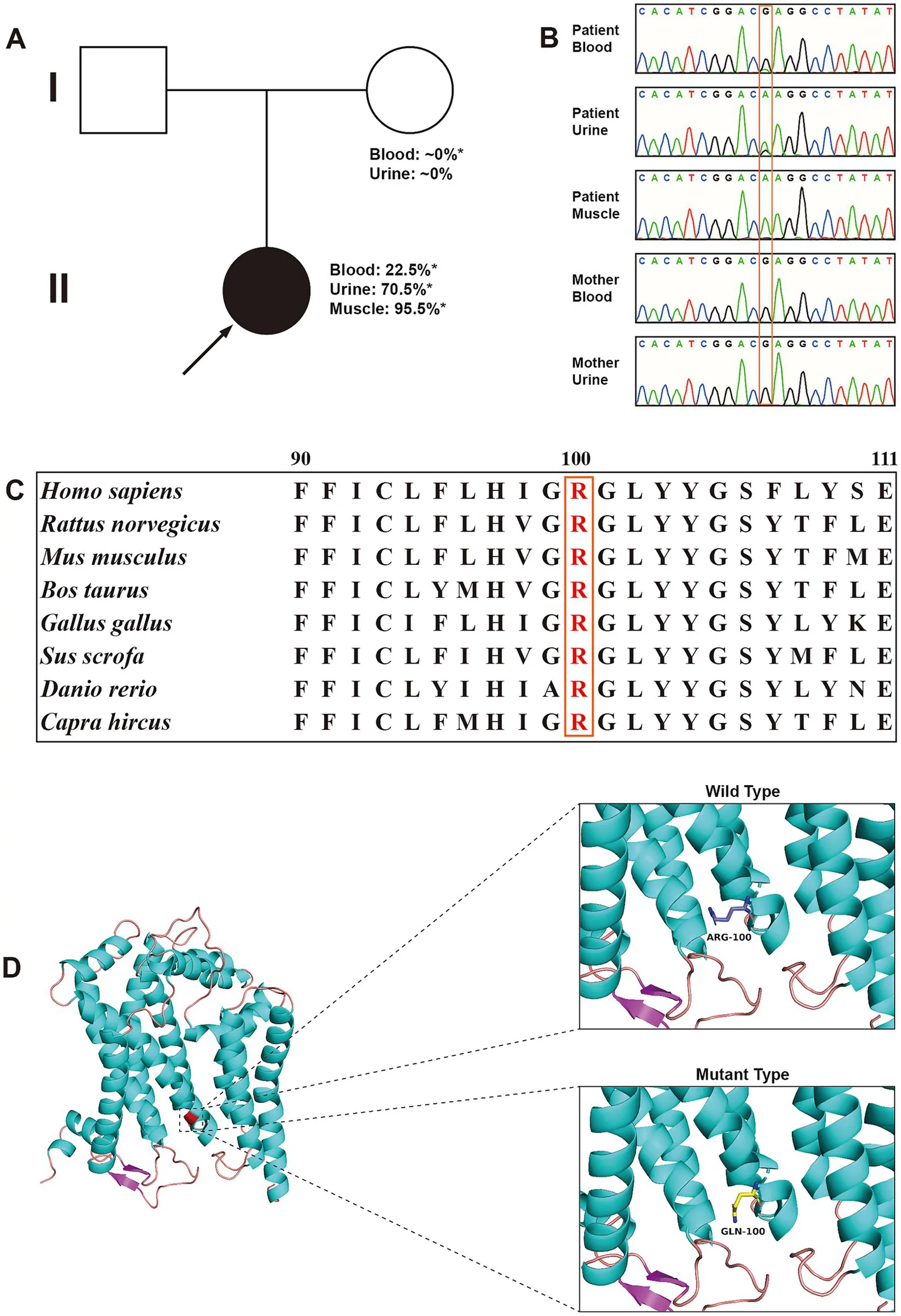
Genetic and structural characterization of the *MT-CYB* variant. **(A)** Family pedigree showing the distribution of the *MT-CYB* variant and tissue-specific heteroplasmy levels. Squares indicate males and circles indicate females. The arrow indicates the proband. Filled symbols represent the affected individual. Heteroplasmy levels of the *MT-CYB* variant in blood, urine, and skeletal muscle are shown. Asterisks indicate heteroplasmy levels determined by next-generation sequencing (NGS). The maternal blood sample was analyzed by NGS, whereas the maternal urine sample was analyzed by Sanger sequencing. **(B)** Sanger sequencing validation of the *MT-CYB* variant in different tissues. The variant was detected in the proband’s blood, urine, and skeletal muscle, but was absent in the mother’s blood and urine. Red boxes indicate the variant position. **(C)** Evolutionary conservation analysis of the *MT-CYB* variant. Multiple sequence alignment of MT-CYB orthologs from different species showing the high conservation of Arg100, which is highlighted in red. **(D)** Structural modeling of wild-type and mutant MT-CYB proteins. Three-dimensional structural models of wild-type and p. Arg100Gln mutant MT-CYB proteins showing the local environment surrounding residue 100.

### Conservation and structural analysis

Multiple sequence alignment of MT-CYB orthologs from different species demonstrated that Arg100 was highly conserved across evolution, suggesting the functional importance of this residue (Figure 3C). Structural modeling of the wild-type and p. Arg100Gln mutant MT-CYB proteins revealed subtle changes in the local structural environment around residue 100. Although the overall hydrogen-bond interaction pattern was largely preserved, the substitution of arginine by glutamine resulted in changes in residue charge properties and side-chain characteristics at this position (Figure 3D).

### Literature review of reported *MT-CYB* variants

A literature review was performed to summarize the clinical, genetic, and biochemical characteristics of reported *MT-CYB* variants (Table 1). A total of 34 individuals, including the present case, carrying *MT-CYB* variants were included in the analysis. The reported clinical phenotypes were heterogeneous, including mitochondrial myopathy (MM, 50.0%), NSMD (26.5%), mitochondrial cardiomyopathy (MCM, 8.8%), MELAS (8.8%), LS (2.9%), and LHON (2.9%) (Figure 4A). Exercise intolerance was the most common initial manifestation, particularly among patients with MM. Other initial presentations included cardiomyopathy, metabolic acidosis, developmental delay, hypotonia, hearing loss, epilepsy, stroke-like episodes, and other less frequent manifestations. The age at onset ranged from infancy to adulthood, with variable age distributions across phenotypes (Figure 4B).

**Table 1:** Clinical, genetic, and biochemical characteristics of individuals with disease-associated *MT-CYB* variants.

No. of patient phenotype	Sex/age at onset	Variant	Heteroplasmy/transmission	Clinical features	Brain MRI/MRS	Lactate/ metabolite	Muscle histology/ RC enzyme activity	Course/ outcome	Reference
1. MM	M/10y	m.15615G > A (p. G290D)	80% (Mu), 0% (B)/ *De novo*	EI, EIM	U/U	4.8↑ (B)/N	RRF (COX+)/CIII↓ (Mu)	Stable/Alive (29y)	(Dumoulin et al., 1996; Mousson et al., 1995)
2. MM	F/25y	m.15762G > A (p. G339E)	85% (Mu), 0% (B)/ De novo	EI, MW	U/U	N/U	N/CIII↓ (Mu)	Stable/Alive (38y)	(Andreu et al., 1998)
3. MM	M/12y	m.15059G > A (p. G105*)	63% (Mu), 0% (B)/ De novo	EI, MW, EIM, MU	U/U	3.9↑ (B)/U	RRF (COX+)/CIII↓ (Mu)	Stable/Alive (27y)	(Andreu et al., 1999)
4. MCM	F/5y	m.15243G > A (p. G166E)	90% (Ht), 100% (F)/ De novo	HCM, MA, HF, LFT↑	N/U	5 ↑ (B)/N	U/CIII↓ (Ht)	Progression/ Dead (9y)	(Valnot et al., 1999)
5. MELAS/JP	M/6y	m.14787_14790del (p. I14fs*50)	95% (Mu), 60% (B, F, H, OM)/De novo	EI, IM, Myo, EP, SE, WPW, Psych, Cortical blindness, Akinetic rigid syndrome	Cortical lesions, Cerebral and cerebellar atrophy/U	2.9↑ (B), 9.8↑ (CSF)/U	Rare RRF/CIII↓ (Mu)	Progression/ Alive (20y)	(De Coo et al., 1999)
6. MM	M/30y	m.15498_15521del (p. G251_L258del)	50% (Mu), 0% (B, F)/U	EI, MW, EIM, MU	U/U	N/U	RRF (COX+)/CIII↓ (Mu)	Stable/Alive (43y)	(Andreu et al., 1999)
7. MM	F/Child	m.14846G > A (p. G34S)	85% (Mu), 0% (B, F)/U	EI, MW, EIM	U/U	↑ (B)/U	RRF (COX+)/CIII↓ (Mu)	Stable/Alive (52y)	
8. MM	F/Child	m.15168G > A (p. W141*)	70% (Mu), 0% (B)/U	EI, EIM, WPW	U/U	↑ (B)/U	RRF (COX+)/CIII↓ (Mu)	Stable/Alive (38y)	
9. MM	M/Child	m.15084G > A (p. W113*)	87% (Mu), 0% (B)/U	EI, EIM	U/U	↑ (B)/U	RRF (COX+)/CIII↓ (Mu)	Stable/Alive (32y)	
10. MM	M/Child	m.15723G > A (p. W326*)	70% (Mu)/U	EI, MW, EIM	U/U	↑ (B)/U	RRF (COX+)/CIII↓ (Mu)	Stable/Alive (51y)	
11. MCM	F/3w	m.15498G > A (p. G251D)	90% (L), 82% (K)/ De novo	Histiocytoid CM, CA, FTT, ATN, Tachypnea, Hepatic steatosis	U/U	U/U	N/CIII↓ (Ht)	Progression/ Dead (1 m)	(Andreu et al., 2000; Papadimitriou et al., 1984)
12. NSMD	F/9y	m.15242G > A (p. G166*)	87% (Mu), 0.7% (B), 4.6% (H), 5% (Ov)/ De novo	EI, MA, EP, Psych, Myo, Diplopia, Stroke, Encephalopathy	U/Pcr↓	↑ (B)/U	RRF (COX+)/CIII↓ (Mu)	Progression/ Alive (34y)	(Keightley et al., 2000)
13. NSMD	F/6y	m.15579A > G (p. Y278C)	88% (Mu), 15% (B)/ De novo	EI, MW, CogI, FTT, HA, EP, RP, Cataract, Deafness	N/U	8↑ (B), 8.8↑ (CSF)/U	RRF (COX+)/CIII↓ (Mu)	Progression/ Alive (19y)	(Wibrand et al., 2001)
14. MM	U/Child	m.15150G > A (p. W135*)	60% (Mu), 0% (B, F)/U	EI, MW	U/U	U/U	U/CIII↓ (Mu)	U/U	(Legros et al., 2001)
15. MM	U/Child	m.15197 T > C (p. S151P)	80% (Mu), 0% (B, F)/U	EI, MW	U/U	U/U	U/CIII↓ (Mu)	U/U	
16. NSMD	M/7 m	m.14849 T > C (p. S35P)	69% (Mu), 6% (B), 12% (F)/De novo	DD, CogI, RM, SS, EI, VI, RP, RI, HT, MA, WPW, HCM, Ataxia, Hypotonia	SOD, Cerebellar hypoplasia/U	31↑ (B)/U	N/CIII↓ (Mu)	Progression/ Alive (25y)	(Schuelke et al., 2002)
17. MM	F/10y	m.15800C > T (p. Q352*)	45% (Mu), 0% (B, F, H, OM)/De novo	EI, EIM, MW	U/U	6.5↑ (B)/U	RRF (COX+), Lipid↑/CI, CIII↓ (Mu)	Stable/Alive (24y)	(Lamantea et al., 2002)
18. MM	F/Child	m.15761G > A (p. G339*)	73% (Mu), 0% (B), <1% (Ur)/De novo	EI, EIM, MW	U/U	6.2↑ (B)/U	RRF (COX+)/CIII↓ (Mu)	Stable/Alive (19y)	(Mancuso et al., 2003)
19. MM	F/30y	m.15170G > A (p. G142*)	99% (Mu), 0% (B, F)/U	EI, EIM, MW	U/U	7.2↑ (B)/U	RRF (COX+)/CI, CIII↓ (Mu)	Stable/Alive (40y)	(Bruno et al., 2003)
20. NSMD	F/Teen	m.15699G > C (p. R318P)	88% (Mu), 13% (B), 42% (F), 16% (Ur)/ De novo	HA, EI, EIM, MW, TIA, SNHL	BGS, CWM lesions/U	3.1↑ (B), 3.9↑ (CSF)/U	RRF (COX+)/CI, CIII↓ (Mu)	Progression/ Alive (38y)	(Blakely et al., 2005)
21. NSMD	M/Birth	m.15635 T > C (p. S297P)	100% (Mu, B, F, L)/ De novo	RD, MA, LF, HCM, MOF, Hypotonia	U/U	23↑ (B)/U	U/CIII↓ (Liver)	Progression/ Dead (1d)	(Fragaki et al., 2009)
22. MM	M/7y	m.14849 T > C (p. S35P)	85% (Mu)/U	EI, EIM, Facial MW, PE	N/U	3.5↑ (B)/U	RRF (COX+), RBF/CII, CIII↓ (Mu)	Stable/Alive (18y)	(Massie et al., 2010)
23. MELAS	F/10y	m.14864 T > C (p. C40R)	39% (Mu), 32% (B), 14% (H), 57% (Ur)/ De novo	HA, SLE, EP, SE, CogI, GD, VI, EI, MW, PN, IM, SS	Temporo-occipital cortical lesions/Lac↑	3.3↑ (B)/U	Subsarcolemmal staining↑/CI, CIII↓ (Mu)	Stable/Alive (15y)	(Emmanuele et al., 2013)
24. MELAS	F/13y	m.15092G > A (p. G116S)	97% (Mu), 45% (B), 87% (Ur)/De novo	HA, SLE, EP, EPC, CogI, VI, PN, Dysphagia	Multifocal cortical lesions/U	↑ (B, CSF)/U	N/CIII↓ (Mu)	Progression/ Alive (21y)	(Mancuso et al., 2014)
25. NSMD	F/Child	m.15649_15666del (p. I302_P307del)	60% (Mu), 7% (B), 65% (F), 36% (Ur)/De novo	EI, MW, GD, RP, VI, OP, PN, DCM, SNHL, Cataract, Hypotonia, Dysphagia, Gastroparesis	Global brain atrophy, CWM lesions/Lac↑, Pcr↓	2.3↑ (B)/U	RRF (COX+)/CI, CIII, CIV↓ (Cybrid cell)	Progression/ Alive (41y)	(Carossa et al., 2014)
26. NSMD	M/7y	m.15319_15327del (p. L192_T194del)	90% (Mu), 0% (B, L), Low heteroplasmy (F)/U	EI, MA, KH, CK↑, LFT↑, Weight loss	N/Lac↑	11↑ (B), 3.1↑ (CSF)/TC↓ (B), LCAC↑ (F)	RRF/CIII↓ (Mu)	Improvement/Alive (15y)	(Mori et al., 2015)
27. NSMD	F/Infant	m.15385_15405del (p. H215_F221del)	72% (Mu), 0% (B)/U	MA, CogI, MW, IM, RP, Blind, Cataract, Spastic tetraparesis, Glaucoma	MCP lesions, Cerebral and cerebellar atrophy/U	2.6↑ (B)/U	RRF/CI, CI + CIII, CII + CIII↓ (Mu)	Progression/ Dead (45y)	(Chaussenot et al., 2018)
28. MM	U/8y	m.15579A > G (p. Y278C)	95% (Mu), Low heteroplasmy (B, OM)/De novo	EI, EIM	U/U	2.4–6↑ (B)/MCAC, LCAC↑ (B)	RRF (COX+)/CIII↓ (Mu)	Stable/Alive (25y)	(Kaphan et al., 2018)
29. MM	M/11y	m.15045G > A (p. R100Q)	100% (Mu), 0% (B, Ur)/U	EI, EIM, PN, Deafness	U/U	2.4–6↑ (B)/ All-chain AC↑ (B)	RRF (COX+)/CIII↓ (Mu)	Stable/Alive (66y)	
30. NSMD	F/1.5y	m.15108 T > G (p. L121R)	30% (Mu), 45% (B), 60% (Ur)/U	DD, EI, MW, GD, ON, FTT, CogI, AHS, Scoliosis, Ptosis, Hypotonia	N/U	3↑ (B)/N	RRF (COX+)/CI, CIII, CIV↓ (Mu)	Progression/ Alive (9y)	(Barcia et al., 2020)
31. MCM	M/U	m.14757 T > C (p. M4T)	U/U	DCM	U/U	U/U	N/CIII↓ (Mu)	U/Alive (40y)	(Zarrouk et al., 2021)
32. LHON	M/19y	m.15309 T > C (p. I188T)	65% (B)/Maternal	ON, Fluctuating VI	N/U	U/U	U/U	Improvement/Alive (27y)	(Petrovic Pajic et al., 2025)
33. LS	M/8 m	m.15635 T > C (p. S297P)	95.0% (Mu), 45.9% (B), 92.6% (Ur)/Maternal	DD, CogI, GD, SNHL, ON, EI, RP, PE, HCM, Dystonia, Dysphagia	BG lesions, Cerebellar atrophy/U	2.8↑ (B)/U	N/CIII↓ (Mu)	Progression/ Alive (45y)	(Tropeano et al., 2025)
34. MM	F/8y	m.15045G > A (p. R100Q)	95.5% (Mu), 22.5% (B), 70.5% (Ur)/De novo	EI, CogI, FTT, SS	N/U	4.8–6.4↑ (B)/ MCAC, LCAC↑ (B)	RRF (COX+)/CIII↓ (Mu)	Stable/Alive (13y)	This study

For plasma/CSF lactate, the unit is mmol/L. The reference range for normality was adopted as reported in the respective source literature. For cases without available exact age at onset, categorical age descriptors reported in the original publications (Birth, Infant, Child, and Teen) were used. NSMD, non-syndromic mitochondrial disease; MM, mitochondrial myopathy; MCM, mitochondrial cardiomyopathy; MELAS, mitochondrial encephalomyopathy with lactate acidosis and stroke-like episodes; LHON, Leber hereditary optic neuropathy; JP, juvenile Parkinsonism; M, male; F, female; w, weeks; m, months; y, years; B, blood; Mu, muscle; F, fibroblasts; Ur, urine; H, hair; OM, oral mucosa; L, liver; K, kidney; Ov, ovary; Ht, heart; CSF, cerebrospinal fluid; +, positive/abnormal finding; −, negative/normal finding; U, unknown/not determined; N, normal; ↑, increased; ↓, decreased; HA, headache; GD, gait disturbance; DD, developmental delay; CogI, cognitive impairment; SLE, stroke-like episodes; TIA, transient ischemic attack; IM, involuntary movement; Myo, myoclonus; EP, epilepsy; EPC, epilepsia partialis continua; SE, status epilepticus; PN, peripheral neuropathy; EI, exercise intolerance; EIM, exercise-induced myalgia; MW, muscle weakness; MU, myoglobinuria; RM, rhabdomyolysis; RP, retinitis pigmentosa; VI, visual impairment; ON, optic neuropathy; OP, ophthalmoplegia; SNHL, sensorineural hearing loss; SS, short stature; FTT, failure to thrive; CM, cardiomyopathy; HCM, hypertrophic cardiomyopathy; DCM, dilated cardiomyopathy; WPW, Wolff-Parkinson-White syndrome; CA, cardiac arrest; HF, heart failure; RD, respiratory distress; AHS, alveolar hypoventilation syndrome; LFT, liver function test; LF, liver failure; RI, renal impairment; ATN, acute tubular necrosis; MA, metabolic acidosis; MOF, multiple organ failure; HT, hypothyroidism; KH, ketotic hypoglycemia; CK, creatine phosphokinase; PE, psychotic episode; Psych, psychiatric symptoms; MRI, magnetic resonance imaging; MRS, magnetic resonance spectroscopy; SOD, septo-optic dysplasia; BG, basal ganglia; BGS, basal ganglia calcification; CWM, cerebral white matter; MCP, middle cerebellar peduncle; PCr, phosphocreatine; Lac, lactate peak; TC, total carnitine; AC, acylcarnitine; MCAC, medium-chain acylcarnitine; LCAC, long-chain acylcarnitine; RRF, ragged-red fiber; COX, cytochrome c oxidase; RC, respiratory chain; CI, complex I; CII, complex II; CIII, complex III; CIV, complex IV.

**Figure 4:**
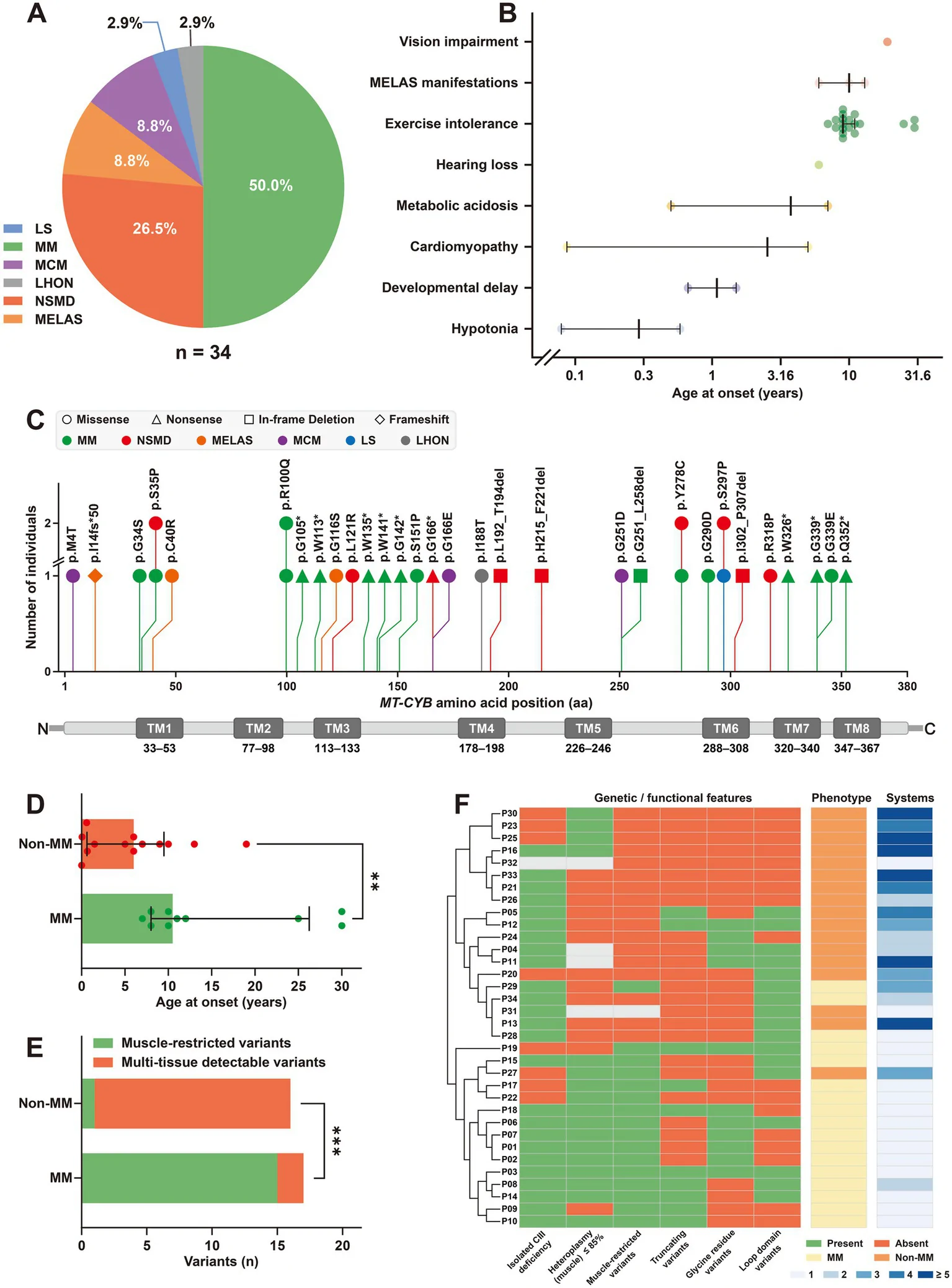
Clinical spectrum and genotype–phenotype correlation of *MT-CYB* variants. **(A)** Clinical phenotype distribution among 34 individuals carrying *MT-CYB* variants. **(B)** Age-at-onset distribution by initial symptoms. Age at onset is presented as median (25th–75th percentiles). The x-axis is displayed on a base-10 logarithmic scale. MELAS-spectrum manifestations include epilepsy, stroke-like episodes, and motor incoordination. When only categorical age-at-onset data were available, representative ages corresponding to the midpoint of each predefined age category were assigned for visualization (e.g., infant = 6 months, child = 9 years, and late childhood = 11 years). **(C)** Distribution of *MT-CYB* variants across the cytochrome b protein. Variant types are indicated by different symbols, and clinical phenotypes are indicated by different colors. Transmembrane domains (TM1–TM8) are shown below the protein schematic. **(D)** Comparison of age at onset between patients with mitochondrial myopathy (MM) and non-MM phenotypes. Age at onset is presented as individual values, with bars indicating the median and 25th–75th percentiles. Statistical comparison was performed using the Mann–Whitney U test. **(E)** Comparison of tissue distribution of *MT-CYB* variants between MM and non-MM phenotypes. Variants were classified as muscle-restricted variants or multi-tissue detectable variants according to the presence or absence of the variant in extra-muscular tissues. Statistical comparison was performed using Fisher’s exact test. **(F)** Hierarchical clustering of clinical, genetic, and functional characteristics of individuals with *MT-CYB* variants. Rows represent individual patients, and columns represent genetic and functional features, clinical phenotype, and number of involved systems. The right-side annotations indicate phenotype classification (MM vs. non-MM) and the number of involved systems. MM, mitochondrial myopathy; NSMD, non-syndromic mitochondrial disease; MCM, mitochondrial cardiomyopathy; MELAS, mitochondrial encephalomyopathy, lactic acidosis, and stroke-like episodes; LS, Leigh syndrome; LHON, Leber hereditary optic neuropathy; TM, transmembrane domain; CIII, complex III.

Among the identified variants, missense variants were the most frequent (20/34, 58.8%), followed by nonsense variants (9/34, 26.5%), in-frame deletions (4/34, 11.8%), and frameshift variants (1/34, 2.9%). Among the 20 patients with available inheritance information, 18 (90.0%) harbored *de novo* variants, whereas 2 (10.0%) showed maternal inheritance. Variants were distributed throughout the cytochrome *b* protein sequence, including different transmembrane domains, without an obvious clustering pattern (Figure 4C). Heteroplasmy levels of *MT-CYB* variants in skeletal muscle varied considerably among reported patients, with a median level of 85% (P25–P75, 67.5–95%; range, 30–100%). Among the 33 patients with available tissue-specific heteroplasmy data, 30 (90.9%) had data from at least two tissues; skeletal muscle and blood heteroplasmy data were available for 30 and 29 patients, respectively (Supplementary Table S7). Sixteen (48.5%) patients showed variants restricted to skeletal muscle, whereas 17 (51.5%) had detectable variants in extra-muscular tissues. Biochemical analysis revealed that 25/33 (75.8%) patients exhibited isolated CIII deficiency, whereas 8/33 (24.2%) showed additional respiratory chain deficiencies involving other complexes, such as CI.

### Genotype–phenotype correlation of *MT-CYB* variants

Genotype–phenotype correlation analysis identified distinct clinical and genetic characteristics distinguishing MM from non-MM phenotypes (Table 2). Compared with patients with non-MM phenotypes, those with MM showed a significantly later age at onset [10.5 (8, 26.25) vs. 6 (0.625, 9.5) years, *p* = 0.007; Figure 4D]. Muscle-restricted *MT-CYB* variants were also significantly more frequent in the MM group (88.2% vs. 6.3%, *p* < 0.001; Figure 4E). In contrast, patients with non-MM phenotypes exhibited involvement of a greater number of organ systems [4 (2, 5) vs. 1 (1, 1), *p* < 0.001]. No significant differences were observed between the two groups with respect to sex, inheritance pattern, variant type, muscle heteroplasmy level, or isolated CIII deficiency (all *p* > 0.05). Hierarchical clustering integrating clinical, genetic, and functional characteristics showed an overall concordance of 88.2% (30/34) with the predefined clinical phenotype classification (MM versus non-MM), correctly classifying 14 of 17 MM cases (82.4%) and 16 of 17 non-MM cases (94.1%). MM cases were predominantly characterized by muscle-restricted variants, isolated CIII deficiency, and single-system involvement, whereas non-MM cases more frequently exhibited variants detectable in extra-muscular tissues, multisystem involvement, and a higher proportion of muscle heteroplasmy >85%. Variant type showed no obvious contribution to cluster separation (Figure 4F).

**Table 2:** Comparison of characteristics between mitochondrial myopathy (MM) and non-MM phenotypes.

Characteristics	MM	Non-MM	*p* value
Male	7/14 (50.00%)	7/17 (41.18%)	0.725
Age at onset (years)	10.5 [8, 26.25]	6 [0.625, 9.5]	0.007^**^
Number of involved systems	1 [1, 1]	4 [2, 5]	< 0.001^***^
De novo variants	7/7 (100.00%)	11/13 (84.62%)	0.521
Truncating variants	8/17 (47.06%)	2/17 (11.76%)	0.057
Missense variants	8/17 (47.06%)	12/17 (70.59%)	0.296
Glycine residue variants^a^	7/17 (41.18%)	4/17 (23.53%)	0.465
Transmembrane domain variants	8/17 (47.06%)	9/17 (52.94%)	1.000
Nucleotide substitution variants	16/17 (94.12%)	13/17 (76.47%)	0.335
Muscle-restricted variants	15/17 (88.24%)	1/16 (6.25%)	< 0.001^***^
Muscle heteroplasmy level	80 [66.5, 91]	88 [64.5, 95]	0.615
Muscle heteroplasmy level > 85%	5/17 (29.41%)	8/13 (61.54%)	0.138
Isolated CIII deficiency	14/17 (82.35%)	11/16 (68.75%)	0.438

^a^Glycine residue variants were defined as variants affecting at least one glycine residue. Data are presented as median [25th percentile, 75th percentile] or n/*N* (%), as appropriate. Continuous variables were compared using the Mann–Whitney *U* test, and categorical variables were compared using Fisher’s exact test. Asterisks indicate statistical significance (^*^p < 0.05, ^**^p < 0.01, ^***^p < 0.001).

MM, mitochondrial myopathy; CIII, complex III.

## Discussion

This study reports a case of childhood-onset MM caused by the *MT-CYB* m.15045G > A variant. This variant was previously considered a polymorphic variant. Kaphan et al. first reported its potential pathogenic role in an adult patient with mitochondrial myopathy, albeit without fully establishing its pathogenicity at that time (Kaphan et al., 2018). Through comprehensive clinical, biochemical, metabolic, and mitochondrial functional assessments of our patient, we further confirm the pathogenicity of the m.15045G > A variant and expand the clinical and variant spectrum of *MT-CYB*-related mitochondrial myopathy. Notably, in contrast to the patient described by Kaphan et al., who presented with exercise intolerance alone, our patient also exhibits borderline intellectual functioning. In the present case, the variant was detectable in both blood and urine, suggesting a broader tissue distribution and raising the possibility that its presence in neural tissue may contribute to this cognitive phenotype, although neural tissue was not available for assessment. In addition, systemic metabolic disturbances secondary to mitochondrial dysfunction may also contribute to central nervous system involvement. In our patient, persistent hyperlactatemia and secondary impairment of fatty acid oxidation, may indirectly affect neuronal function. In particular, medium-chain acylcarnitines have been shown experimentally to induce oxidative stress in brain tissue, suggesting a potential mechanism through which accumulated metabolites may contribute to neurological dysfunction (Wajner and Amaral, 2015). Therefore, both potential variant involvement in neural tissue and secondary systemic metabolic disturbances may contribute to the mild cognitive phenotype in our patient, although their relative contributions remain uncertain.

Mitochondrial cytochrome b is a critical component of the catalytic core of CIII. It contains two prosthetic groups, heme bL and heme bH, which are positioned on opposite sides of the inner mitochondrial membrane and play a critical role in transmembrane electron transfer within CIII (Iwata et al., 1998; Bazil, 2017). The m.15045G > A variant results in a p. Arg100Gln substitution at a highly conserved residue within a transmembrane helix of cytochrome b. Given that arginine residues often play key roles in maintaining local electrostatic environments, participating in proton translocation across membranes, or facilitating specific interactions with ligands (Li et al., 2008; Ding et al., 1995), this variant may indirectly affect the conformational stability of *MT-CYB* or the electron transfer activity of CIII by altering local electrostatic potentials, side-chain steric hindrance, or non-covalent interactions with surrounding functional residues. Mitochondrial functional analysis of our patient confirmed significantly reduced CIII activity in muscle tissue, providing direct biochemical evidence for the pathogenicity of this variant. Notably, the heteroplasmy level in cultured fibroblasts was approximately 35%, substantially lower than that in skeletal muscle. This lower heteroplasmy may partly account for the relatively preserved mitochondrial respiratory function in fibroblasts. In addition, as only one healthy control cell line was included in the fibroblast-based mitochondrial respiration analysis, inter-individual biological variability among healthy individuals should also be considered when interpreting these findings.

In terms of metabolic features, our patient exhibited persistent elevations of medium and long-chain acylcarnitines, representing a metabolic profile mimicking multiple acyl-CoA dehydrogenase deficiency (MADD). Mitochondrial CIII oxidizes ubiquinol back to ubiquinone, thereby maintaining the availability of CoQ for continuous electron transfer from multiple mitochondrial pathways, including fatty acid β-oxidation via the electron transfer flavoprotein (ETF)/electron transfer flavoprotein dehydrogenase (ETFDH) systems (Banerjee et al., 2022; Martínez-Reyes et al., 2020). Therefore, CIII deficiency may secondarily impair fatty acid oxidation by disrupting electron flow through this pathway, leading to accumulation of acylcarnitine species. Interestingly, accumulation of medium-chain acyl-CoAs, such as octanoyl-CoA, has been shown to inhibit CIII activity by impairing cytochrome b reduction (Sauer et al., 2008), suggesting a potential vicious cycle between CIII dysfunction and secondary metabolic disturbances that may further exacerbate mitochondrial dysfunction. Although metabolic profiling was not systematically performed in most reported patients with *MT-CYB* variants, several cases with available metabolic data have demonstrated similar abnormalities, including elevated medium and long-chain acylcarnitines (Mori et al., 2015; Kaphan et al., 2018). These findings suggest that altered fatty acid metabolism may represent an underrecognized metabolic feature in a subset of patients with *MT-CYB*-related CIII deficiency.

Beyond the findings observed in our patient, we further explored the broader clinical spectrum of *MT-CYB*-associated mitochondrial disease through a comprehensive review of previously reported cases. *MT-CYB* variants represent a rare cause of mitochondrial disease, and only a limited number of pathogenic variants have been reported to date. Historically, *MT-CYB* variants have been primarily recognized as a cause of MM, with exercise intolerance being the most frequently reported manifestation. Consistent with previous observations, our analysis demonstrated that MM represented the predominant clinical phenotype. This predominance is biologically plausible, as cytochrome b is the only mitochondrially encoded subunit of respiratory chain CIII, which is essential for OXPHOS (Crofts, 2004). Consequently, impaired CIII activity preferentially affects skeletal muscle, resulting in exercise intolerance and other myopathic manifestations. However, the clinical presentation is considerably broader than previously appreciated. In addition to isolated myopathy, *MT-CYB* variants have been associated with multisystem disorders, including MELAS-spectrum manifestations, MCM, LS, and LHON. These findings indicate that *MT-CYB*-associated disease should be regarded as a continuous clinical spectrum rather than a single myopathic disorder (Emmanuele et al., 2013). Accordingly, *MT-CYB* variants should be considered in patients presenting with diverse mitochondrial phenotypes, particularly when isolated CIII deficiency is identified. The mechanisms underlying this marked phenotypic heterogeneity warrant further investigation.

A major finding of the present study was the observed association between tissue-specific distribution of *MT-CYB* variants and clinical phenotype. Patients with mitochondrial myopathy were significantly more likely to harbor muscle-restricted *MT-CYB* variants, whereas variants detectable in extra-muscular tissues were predominantly observed in patients with non-myopathic phenotypes. Previous studies similarly reported that *MT-CYB* variants in patients with isolated myopathy were often undetectable in accessible non-muscle tissues, suggesting that these variants may arise *de novo* in muscle progenitor cells after germ-layer differentiation or preferentially segregate to skeletal muscle during early embryogenesis through skewed mitotic segregation or negative selection in non-muscle tissues (Andreu et al., 1999; Keightley et al., 2000). Notably, tissue sampling was not standardized across historical reports, and the number and types of extra-muscular tissues examined were limited in some patients, including two patients with MM for whom only skeletal muscle heteroplasmy was reported. In addition, heteroplasmy detection methods and their analytical sensitivity varied across studies, such that low-level variants in extra-muscular tissues may have remained undetected. Despite these potential biases, the current findings still support this association. Prospective studies using standardized and sensitive multi-tissue heteroplasmy assessment are needed to determine whether it reflects a causal biological relationship. In contrast, skeletal muscle heteroplasmy levels were not significantly associated with clinical phenotype, suggesting that heteroplasmy in a single tissue may not adequately predict the overall clinical phenotype. No clear genotype–phenotype relationship was observed for variant type or biochemical profile. Interestingly, although the difference did not reach statistical significance, truncating variants were more frequent in the MM group, whereas missense variants predominated among patients with more extensive multisystem involvement. This pattern appears counterintuitive, as it contrasts with the general expectation that truncating variants have greater functional consequences, suggesting that variant type alone may not reliably predict clinical phenotype. Phenotypic expression may instead be more closely associated with tissue distribution and tissue-specific functional thresholds. Hierarchical clustering further revealed partially distinct patterns between MM and non-MM, supporting the combined contribution of multiple genetic, biochemical, and tissue-specific factors to phenotypic expression. Although tissue distribution of *MT-CYB* variants showed a more prominent association with phenotype in our analysis, other genetic and functional characteristics may also contribute collectively to phenotypic expression. Given the limited sample size, however, these clustering patterns should be considered exploratory, and further studies in larger, well-characterized cohorts are needed to clarify the relative contributions and interactions of these factors.

The genotype–phenotype associations observed in our cohort may also have practical implications for clinical management. In patients with predominantly myopathic presentations, management may focus on maintaining muscle function and exercise tolerance through individualized aerobic rehabilitation and supportive metabolic therapy, while minimizing metabolic stressors that may precipitate or exacerbate metabolic decompensation (Tarnopolsky and Raha, 2005). In contrast, patients with non-MM phenotypes frequently exhibit multisystem involvement and may therefore require broader and more intensive surveillance, particularly for neurological and cardiac complications, including encephalopathy, seizures, stroke-like episodes, and cardiomyopathy. Regular assessment of other potentially affected systems should also be considered according to the individual phenotype. Importantly, despite the observed association between tissue-specific variant distribution and clinical phenotype, these management considerations should remain primarily phenotype-oriented rather than genotype-specific, with longitudinal surveillance to identify evolving or newly emerging organ involvement. As the present study did not evaluate treatment efficacy across the two subgroups, future prospective studies evaluating phenotype-stratified management strategies may provide valuable insights into optimizing clinical care and improving outcomes in patients with *MT-CYB*-associated mitochondrial disease.

### Limitations

Several limitations should be acknowledged. First, although biochemical and functional analyses supported the pathogenicity of the m.15045G > A variant, further studies are needed to clarify its molecular effects on CIII function. Second, the rarity of *MT-CYB* variants resulted in a relatively small sample size, which limited statistical power, particularly for subgroup and genotype–phenotype analyses. Third, several historically reported disease-associated *MT-CYB* variants are currently classified as VUS under contemporary mtDNA-specific ACMG/AMP criteria. Although these variants had published evidence supporting a potential disease-causing role, uncertainty regarding their pathogenicity may have introduced heterogeneity into the genotype–phenotype analyses. Fourth, the retrospective nature of the literature review introduced heterogeneity in clinical evaluation, tissue sampling, heteroplasmy measurement, and biochemical assessment across reported cases, with incomplete data for some variables. In particular, differences in diagnostic tissue sampling according to clinical presentation may have introduced ascertainment bias, while variation in heteroplasmy detection methods may have introduced measurement bias, potentially influencing the observed association between tissue distribution and clinical phenotype. Finally, the hierarchical clustering analysis was exploratory and requires validation in larger, well-characterized cohorts before robust genotype–phenotype relationships can be established.

## Conclusion

Our study provides further evidence for the pathogenicity of the *MT-CYB* m.15045G > A variant and expands the clinical and metabolic spectrum of *MT-CYB*-associated mitochondrial disease. Comprehensive analysis highlights its broad phenotypic heterogeneity and identifies an association between the tissue distribution of *MT-CYB* variants and clinical phenotype, with muscle-restricted variants more frequently observed in patients with mitochondrial myopathy, while other genetic and functional characteristics may also contribute to phenotypic expression.

## Data Availability

The datasets presented in this article are not readily available because the original data that support the findings of this study are available from the corresponding author upon reasonable request. Requests to access the datasets should be directed to FF, fangfang@bch.com.cn.

## References

[ref1] Andreu A. L. Bruno C. Dunne T. C. Tanji K. Shanske S. Sue C. M. . (1999). A nonsense mutation (G15059A) in the cytochrome b gene in a patient with exercise intolerance and myoglobinuria. Ann. Neurol. 45, 127–130. doi: 10.1002/1531-8249(199901)45:1<127::AID-ART20>3.0.CO;2-Y, 9894887

[ref2] Andreu A. L. Bruno C. Shanske S. Shtilbans A. Hirano M. Krishna S. . (1998). Missense mutation in the mtDNA cytochrome b gene in a patient with myopathy. Neurology 51, 1444–1447. doi: 10.1212/WNL.51.5.1444, 9818877

[ref3] Andreu A. L. Checcarelli N. Iwata S. Shanske S. Dimauro S. (2000). A missense mutation in the mitochondrial cytochrome b gene in a revisited case with histiocytoid cardiomyopathy. Pediatr. Res. 48, 311–314. doi: 10.1203/00006450-200009000-00008, 10960495

[ref4] Andreu A. L. Hanna M. G. Reichmann H. Bruno C. Penn A. S. Tanji K. . (1999). Exercise intolerance due to mutations in the cytochrome b gene of mitochondrial DNA. N. Engl. J. Med. 341, 1037–1044. doi: 10.1056/NEJM199909303411404, 10502593

[ref5] Banerjee R. Purhonen J. Kallijärvi J. (2022). The mitochondrial coenzyme Q junction and complex III: biochemistry and pathophysiology. FEBS J. 289, 6936–6958. doi: 10.1111/febs.16164, 34428349

[ref6] Barcia G. Khirani S. Amaddeo A. Assouline Z. Pennisi A. Boddaert N. . (2020). Evidence of diaphragmatic dysfunction with severe alveolar hypoventilation syndrome in mitochondrial respiratory chain deficiency. Neuromuscular Dis 30, 593–598. doi: 10.1016/j.nmd.2020.06.002, 32654952

[ref7] Bazil J. N. (2017). Analysis of a functional dimer Model of Ubiquinol cytochrome c oxidoreductase. Biophys. J. 113, 1599–1612. doi: 10.1016/j.bpj.2017.08.018, 28978450 PMC5627346

[ref8] Bénit P. Lebon S. Rustin P. (2009). Respiratory-chain diseases related to complex III deficiency. Biochim. Biophys. Acta 1793, 181–185. doi: 10.1016/j.bbamcr.2008.06.004, 18601960

[ref9] Bernier F. P. Boneh A. Dennett X. Chow C. W. Cleary M. A. Thorburn D. R. (2002). Diagnostic criteria for respiratory chain disorders in adults and children. Neurology 59, 1406–1411. doi: 10.1212/01.WNL.0000033795.17156.00, 12427892

[ref10] Blakely E. L. Mitchell A. L. Fisher N. Meunier B. Nijtmans L. G. Schaefer A. M. . (2005). A mitochondrial cytochrome b mutation causing severe respiratory chain enzyme deficiency in humans and yeast. FEBS J. 272, 3583–3592. doi: 10.1111/j.1742-4658.2005.04779.x, 16008558

[ref11] Bruno C. Santorelli F. M. Assereto S. Tonoli E. Tessa A. Traverso M. . (2003). Progressive exercise intolerance associated with a new muscle-restricted nonsense mutation (G142X) in the mitochondrial cytochrome b gene. Muscle Nerve 28, 508–511. doi: 10.1002/mus.10429, 14506725

[ref12] Carossa V. Ghelli A. Tropeano C. V. Valentino M. L. Iommarini L. Maresca A. . (2014). A novel in-frame 18-bp microdeletion in MT-CYB causes a multisystem disorder with prominent exercise intolerance. Hum. Mutat. 35, 954–958. doi: 10.1002/humu.22596, 24863938

[ref13] Chaussenot A. Rouzier C. Fragaki K. Sacconi S. Ait-el-Mkadem S. Paquis-Flucklinger V. . (2018). MT-CYB deletion in an encephalomyopathy with hyperintensity of middle cerebellar peduncles. Neurology Genetics 4:e268. doi: 10.1212/NXG.0000000000000268, 30294674 PMC6168751

[ref14] Crofts A. R. (2004). The cytochrome bc1 complex: function in the context of structure. Annu. Rev. Physiol. 66, 689–733. doi: 10.1146/annurev.physiol.66.032102.150251, 14977419

[ref15] De Coo I. F. Renier W. O. Ruitenbeek W. De Coo I. F. M. Ter Laak H. J. Bakker M. . (1999). A 4-base pair deletion in the mitochondrial cytochrome b gene associated with parkinsonism/MELAS overlap syndrome. Ann. Neurol. 45, 130–133. doi: 10.1002/1531-8249(199901)45:1<>3.3.co;2-q, 9894888

[ref16] Ding H. Daldal F. Dutton P. L. (1995). Ion pair formation between basic residues at 144 of the Cyt b polypeptide and the ubiquinones at the Qo site of the Cyt bc1 complex. Biochemistry 34, 15997–16003. doi: 10.1021/bi00049a013, 8519755

[ref17] Dumoulin R. Sagnol I. Ferlin T. Bozon D. Stepien G. Mousson B. (1996). A novel gly290asp mitochondrial cytochrome b mutation linked to a complex III deficiency in progressive exercise intolerance. Mol. Cell. Probes 10, 389–391. doi: 10.1006/mcpr.1996.0053, 8910895

[ref18] Emmanuele V. Sotiriou E. Rios P. G. Ganesh J. Ichord R. Foley A. R. . (2013). A novel mutation in the mitochondrial DNA cytochrome b gene (MTCYB) in a patient with mitochondrial encephalomyopathy, lactic acidosis, and strokelike episodes syndrome. J. Child Neurol. 28, 236–242. doi: 10.1177/0883073812445787, 22638077 PMC3973035

[ref19] Fragaki K. Procaccio V. Bannwarth S. Serre V. O’Hearn S. Potluri P. . (2009). A neonatal polyvisceral failure linked to a de novo homoplasmic mutation in the mitochondrially encoded cytochrome b gene. Mitochondrion 9, 346–352. doi: 10.1016/j.mito.2009.06.002, 19563916

[ref20] Hagen C. M. Aidt F. H. Havndrup O. Hedley P. L. Jespersgaard C. Jensen M. . (2013). MT-CYB mutations in hypertrophic cardiomyopathy. Mol. Genet. Genomic Med. 1, 54–65. doi: 10.1002/mgg3.5, 24498601 PMC3893158

[ref21] Higuchi Y. Okunushi R. Hara T. Hashiguchi A. Yuan J. Yoshimura A. . (2018). Mutations in COA7 cause spinocerebellar ataxia with axonal neuropathy. Brain: A J Neurol 141, 1622–1636. doi: 10.1093/brain/awy104, 29718187 PMC5972596

[ref22] Iwata S. Lee J. W. Okada K. Lee J. K. Iwata M. Rasmussen B. . (1998). Complete structure of the 11-subunit bovine mitochondrial cytochrome bc1 complex. Science (New York, N.Y.) 281, 64–71. doi: 10.1126/science.281.5373.64, 9651245

[ref23] Jiang H. Xu C. Liu Z. Duan R. Yao X. Fu X. . (2025). Case report: Biallelic variants in MRPS36, encoding a component of the 2-oxoglutarate dehydrogenase complex, cause leigh syndrome. Front. Pediatr. 13:1608840. doi: 10.3389/fped.2025.1608840, 41018056 PMC12463997

[ref24] Kaphan E. Bou Ali H. Gastaldi M. Acquaviva C. Vianey-Saban C. Rouzier C. . (2018). Myopathy with MTCYB mutation mimicking multiple acyl-CoA dehydrogenase deficiency. Rev. Neurol. 174, 731–735. doi: 10.1016/j.neurol.2018.03.014, 30318261

[ref25] Keightley J. A. Anitori R. Burton M. D. Quan F. Buist N. R. M. Kennaway N. G. (2000). Mitochondrial encephalomyopathy and complex III deficiency associated with a stop-codon mutation in the cytochrome b gene. Am. J. Hum. Genet. 67, 1400–1410. doi: 10.1086/316900, 11047755 PMC1287917

[ref26] Lamantea E. Carrara F. Mariotti C. Morandi L. Tiranti V. Zeviani M. (2002). A novel nonsense mutation (Q352X) in the mitochondrial cytochrome b gene associated with a combined deficiency of complexes I and III. Neuromuscular Dis 12, 49–52. doi: 10.1016/S0960-8966(01)00244-9, 11731284

[ref27] Legros F. Chatzoglou E. Frachon P. Ogier de Baulny H. Laforêt P. Jardel C. . (2001). Functional characterization of novel mutations in the human cytochrome b gene. European journal of human genetics 9, 510–518. doi: 10.1038/sj.ejhg.5200678, 11464242

[ref29] Li L. Vorobyov I. Allen T. W. (2008). Potential of mean force and pKa profile calculation for a lipid membrane-exposed arginine side chain. J. Phys. Chem. B 112, 9574–9587. doi: 10.1021/jp7114912, 18636765

[ref30] Mancuso M. Filosto M. Stevens J. C. Patterson M. Shanske S. Krishna S. . (2003). Mitochondrial myopathy and complex III deficiency in a patient with a new stop-codon mutation (G339X) in the cytochrome b gene. J. Neurol. Sci. 209, 61–63. doi: 10.1016/S0022-510X(02)00462-8, 12686403

[ref31] Mancuso M. Nesti C. Ienco E. C. Orsucci D. Pizzanelli C. Chiti A. . (2014). Novel MTCYB mutation in a young patient with recurrent stroke-like episodes and status epilepticus. Am. J. Med. Genet. A 164a, 2922–2925. doi: 10.1002/ajmg.a.36725, 25125337

[ref32] Martínez-Reyes I. Cardona L. R. Kong H. Vasan K. McElroy G. S. Werner M. . (2020). Mitochondrial ubiquinol oxidation is necessary for tumour growth. Nature 585, 288–292. doi: 10.1038/s41586-020-2475-6, 32641834 PMC7486261

[ref33] Massie R. Wong L. J. Milone M. (2010). Exercise intolerance due to cytochrome b mutation. Muscle Nerve 42, 136–140. doi: 10.1002/mus.21649, 20544923

[ref34] McCormick E. M. Lott M. T. Dulik M. C. Shen L. Attimonelli M. Vitale O. . (2020). Specifications of the ACMG/AMP standards and guidelines for mitochondrial DNA variant interpretation. Hum. Mutat. 41, 2028–2057. doi: 10.1002/humu.24107, 32906214 PMC7717623

[ref35] Mori M. Goldstein J. Young S. P. Bossen E. H. Shoffner J. Koeberl D. D. (2015). Complex III deficiency due to an in-frame MT-CYB deletion presenting as ketotic hypoglycemia and lactic acidosis. Molecular genetics and metabolism reports 4, 39–41. doi: 10.1016/j.ymgmr.2015.06.001, 26937408 PMC4750615

[ref36] Mousson B. Collombet J. M. Dumoulin R. Carrier H. Flocard F. Bouzidi M. . (1995). An abnormal exercise test response revealing a respiratory chain complex III deficiency. Acta Neurol. Scand. 91, 488–493. doi: 10.1111/j.1600-0404.1995.tb00451.x, 7572045

[ref37] Ogawa E. Shimura M. Fushimi T. Tajika M. Ichimoto K. Matsunaga A. . (2017). Clinical validity of biochemical and molecular analysis in diagnosing Leigh syndrome: a study of 106 Japanese patients. J. Inherit. Metab. Dis. 40, 685–693. doi: 10.1007/s10545-017-0042-6, 28429146 PMC5579154

[ref38] Papadimitriou A. Neustein H. B. Dimauro S. Stanton R. Bresolin N. (1984). Histiocytoid cardiomyopathy of infancy: deficiency of reducible cytochrome b in heart mitochondria. Pediatr. Res. 18, 1023–1028. doi: 10.1203/00006450-198418100-00023, 6093033

[ref39] Petrovic Pajic S. Fakin A. Jarc-Vidmar M. Sustar Habjan M. Malinar L. Pavlovic K. . (2025). Atypical Leber hereditary optic neuropathy (LHON) associated with a novel MT-CYB:m.15309T>C(Ile188Thr) variant. Genes 16:108. doi: 10.3390/genes16010108, 39858655 PMC11764998

[ref40] Richards S. Aziz N. Bale S. Bick D. das S. Gastier-Foster J. . (2015). Standards and guidelines for the interpretation of sequence variants: a joint consensus recommendation of the American College of Medical Genetics and Genomics and the Association for Molecular Pathology. Genetics. Med 17, 405–424. doi: 10.1038/gim.2015.30, 25741868 PMC4544753

[ref41] Sauer S. W. Okun J. G. Hoffmann G. F. Koelker S. Morath M. A. (2008). Impact of short and medium-chain organic acids, acylcarnitines, and acyl-CoAs on mitochondrial energy metabolism. Biochim. Biophys. Acta 1777, 1276–1282. doi: 10.1016/j.bbabio.2008.05.447, 18582432

[ref42] Schuelke M. Krude H. Finckh B. Mayatepek E. Janssen A. Schmelz M. . (2002). Septo-optic dysplasia associated with a new mitochondrial cytochrome b mutation. Ann. Neurol. 51, 388–392. doi: 10.1002/ana.10151, 11891837

[ref43] Stewart L. A. Clarke M. Rovers M. Riley R. D. Simmonds M. Stewart G. . (2015). Preferred reporting items for systematic review and meta-analyses of individual participant data: the PRISMA-IPD statement. JAMA 313, 1657–1665. doi: 10.1001/jama.2015.3656 25919529

[ref44] Tarnopolsky M. A. Raha S. (2005). Mitochondrial myopathies: diagnosis, exercise intolerance, and treatment options. Med. Sci. Sports Exerc. 37, 2086–2093. doi: 10.1249/01.mss.0000177341.89478.06, 16331134

[ref46] Tropeano C. V. La Morgia C. Achilli A. Iommarini L. Tioli G. Caporali L. . (2025). Adult Leigh syndrome associated with the m.15635T>C mitochondrial DNA variant affecting the cytochrome b (MT-CYB) gene. Int. J. Mol. Sci. 26:1116. doi: 10.3390/ijms26031116, 39940884 PMC11817157

[ref47] Valnot I. Kassis J. Chretien D. de Lonlay P. Parfait B. Munnich A. . (1999). A mitochondrial cytochrome b mutation but no mutations of nuclearly encoded subunits in ubiquinol cytochrome c reductase (complex III) deficiency. Hum. Genet. 104, 460–466. doi: 10.1007/s004390050988, 10453733

[ref48] Wajner M. Amaral A. U. (2015). Mitochondrial dysfunction in fatty acid oxidation disorders: insights from human and animal studies. Biosci. Rep. 36:e00281. doi: 10.1042/BSR20150240, 26589966 PMC4718505

[ref49] Wibrand F. Ravn K. Schwartz M. Rosenberg T. Horn N. Vissing J. (2001). Multisystem disorder associated with a missense mutation in the mitochondrial cytochrome b gene. Ann. Neurol. 50, 540–543. doi: 10.1002/ana.1224, 11601507

[ref50] Zarrouk S. Finsterer J. Mehri S. Ourda F. Ben Arab S. Boussada R. (2021). Dilated cardiomyopathy due to the novel MT-CYB missense mutation m.14757T>C. J. Med Cases 12, 455–459. doi: 10.14740/jmc3787, 34804306 PMC8577615

