# Supplementary material for: Clinical spectrum and genotype–phenotype correlations of MT-CYB-associated mitochondrial disease: from the m.15045G > A variant to the broader disease landscape
Source: Front Neurosci. 2026 Sep 29;20:1968308. doi: 10.3389/fnins.2026.1968308 (PMC13658607; doi:10.3389/fnins.2026.1968308)
Supplement: Supplementary file 1 [file Table_1.docx]

**Supplementary Table**

**Table S1** Long-term laboratory control/reference data for skeletal muscle mitochondrial respiratory chain enzyme assays

| **Control** | **CI** | **CII** | **CII+III** | **CIII** | **CIV** | **CS** | **CI/CS** | **CII/CS** | **CII+III/CS** | **CIII/CS** | **CIV/CS** | **CI/CII** | **CII+III/CII** | **CIII/CII** | **CIV/CII** |
| --- | --- | --- | --- | --- | --- | --- | --- | --- | --- | --- | --- | --- | --- | --- | --- |
| Reference control* | 64.57 | 59.17 | 55.04 | 32.77 | 5.34 | 186.09 | 345.98 | 312.77 | 299.34 | 171.84 | 30.49 | 1136.73 | 985.90 | 561.00 | 103.19 |
| Control material 2 | 48.90 | 61.29 | 45.96 | 23.40 | 8.74 | 163.97 | 298.23 | 373.79 | 280.30 | 142.71 | 53.30 | 797.85 | 749.88 | 381.79 | 142.60 |
| Control material 3 | 38.28 | 36.16 | 40.92 | 27.38 | 4.55 | 117.22 | 326.57 | 308.48 | 349.09 | 233.58 | 38.82 | 1058.63 | 1131.64 | 757.19 | 125.83 |
| Control material 4 | 98.11 | 72.95 | 103.70 | 50.06 | 10.58 | 207.05 | 473.85 | 352.33 | 500.85 | 241.78 | 51.10 | 1344.89 | 1421.52 | 686.22 | 145.03 |
| Control material 5 | 50.25 | 55.56 | 46.90 | 59.22 | 8.64 | 147.15 | 341.49 | 377.57 | 318.72 | 402.45 | 58.72 | 904.43 | 844.13 | 1065.87 | 155.51 |
| Control material 6 | 62.41 | 67.25 | 55.54 | 69.80 | 8.09 | 175.82 | 354.97 | 382.49 | 315.89 | 397.00 | 46.01 | 928.03 | 825.87 | 1037.92 | 120.30 |
| Control material 7 | 74.42 | 64.72 | 74.77 | 31.77 | 11.76 | 230.94 | 322.25 | 280.25 | 323.76 | 137.57 | 50.92 | 1149.88 | 1155.28 | 490.88 | 181.71 |
| Control material 8 | 25.73 | 42.93 | 41.71 | 23.68 | 6.01 | 116.01 | 221.79 | 370.05 | 359.54 | 204.12 | 51.81 | 599.35 | 971.58 | 551.60 | 140.00 |
| Control material 9 | 48.74 | 71.05 | 62.10 | 55.87 | 10.15 | 190.52 | 255.83 | 372.93 | 325.95 | 293.25 | 53.28 | 686.00 | 874.03 | 786.35 | 142.86 |

**Notes:** * The reference control was repeatedly analyzed over multiple historical assay runs. Summary statistics are presented rather than individual run-level measurements. These laboratory control materials were used for routine monitoring and verification of the assay reference system and should not be interpreted as a cohort of nine healthy individuals. **Abbreviations:** CI–CIV, mitochondrial respiratory chain complexes I–IV; CS, citrate synthase.

**Table S2** Search strategies in PubMed and EMBASE

| Pubmed | ((((MT-CYB) OR (MT CYB)) OR (MTCYB)) OR (Mitochondrial cytochrome b)) AND (((((((((Mutation) OR (Mutations)) OR (Variant)) OR (Variants)) OR (Missense)) OR (Nonsense)) OR (Deletion)) OR (Insertion)) OR (Frameshift)) |
| --- | --- |
| EMBASE | ('MT-CYB'/exp OR 'MT-CYB':ti,ab OR MTCYB:ti,ab OR 'mitochondrial cytochrome b':ti,ab) AND ('mutation'/exp OR 'genetic variant'/exp OR mutation:ti,ab OR variant:ti,ab) |

**Table S3** Studies excluded due to insufficient evidence supporting a disease-causing role of the *MT-CYB* variant

| **No.** | **Article title** | ***MT-CYB* variant** | **PMID** | **Reason for exclusion** |
| --- | --- | --- | --- | --- |
| 1 | Autosomal Dominant Deletions of the Mitochondrial Genome in a Case of Progressive Encephalomyopathy | Multiple mtDNA deletions spanning the *COX II*–*MT-CYB* region | 2014791 | No discrete pathogenic MT-CYB variant identified. Multiple mtDNA deletions were also detected in unaffected maternal relatives, and the authors questioned their causal relationship with the phenotype. |
| 2 | Cytochrome b Mutations in Leber Hereditary Optic Neuropathy | m.15257G>A | 1764087 | The MT-CYB variants frequently co-occurred with other LHON-associated mtDNA variants, particularly m.13708G>A, supporting a possible synergistic/modifying rather than independent primary pathogenic role. |
| 3 | Mitochondrial DNA Complex I and III Mutations Associated With Leber’s Hereditary Optic Neuropathy | m.15257G>A; m.15812G>A | 1732158 | Variants occurred in a complex mtDNA background with m.13708G>A and/or m.5244G>A and were also detected at low frequency in controls; the authors proposed synergistic effects of multiple mtDNA variants rather than isolated MT-CYB causality. |
| 4 | Leber’s Hereditary Optic Neuropathy: Clinical Manifestations of the 15257 Mutation | m.15257G>A | 8321540 | m.15257G>A occurred in pedigrees carrying additional LHON-associated mtDNA variants, preventing attribution of the phenotype to the MT-CYB variant alone. |
| 5 | Leber's optic neuropathy: a case report | m.15257G>A | 16402322 | The patient carried the primary LHON-associated MT-ND4 m.11778G>A variant; m.15257G>A was considered a secondary mtDNA variant insufficient to cause disease independently. |
| 6 | Novel Mutations in Mitochondrial Cytochrome b in Fatal Post Partum Cardiomyopathy | m.15509A>C | 7623448 | Insufficient evidence of pathogenicity. The variant was reported in a single patient, and its heteroplasmy status and population frequency had not been established; the authors stated that further studies were required. |
| 7 | A Point Mutation in the Cytb Gene of Cardiac mtDNA Associated With Complex III Deficiency in Ischemic Cardiomyopathy | m.15452C>A | 8908357 | The variant was detected in controls and studied in patients with ischemic cardiomyopathy/coronary artery disease, supporting an association or susceptibility effect rather than an established primary MT-CYB-related mitochondrial disorder. |
| 8 | A Novel Missense Mutation 15747 T>C in the Mitochondrial Cytochrome b Gene | m.15747T>C | 10571957 | Lack of segregation with disease. The variant was virtually homoplasmic and present in all five family members, including three asymptomatic relatives. |
| 9 | Mitochondrial DNA Mutations in Patients With Orthostatic Hypotension | m.15323A>G | 10449650 | The variant co-occurred with an MT-CO3 variant in the same patient, and the study provided only preliminary association evidence; further studies were explicitly considered necessary. |
| 10 | A Case of Leber Optic Neuropathy With a New Point Mutation in the Cytochrome b Gene | m.14894T>C | 10663786 | The variant was also present in two unaffected maternal relatives, with no direct functional evidence establishing causality; the authors described the association as suggestive. |
| 11 | Mitochondriopathy Mimicking Amyotrophic Lateral Sclerosis | *MT-CYB* His→Leu substitution (nucleotide change not specified) | 12801431 | Multiple mtDNA substitutions involving mt-tRNAIle, MT-ATP6, and MT-CYB were simultaneously present, with no variant-specific functional or segregation evidence to establish MT-CYB as the causal defect. |
| 12 | Extensive and Rapid Screening for Major Mitochondrial DNA Point Mutations in Patients With Hereditary Hearing Loss | m.15498G>A | 20111055 | The variant was detected in a screening cohort, but the study provided insufficient individual-level segregation or functional evidence linking the variant specifically to hearing loss. This is a case-level attribution issue rather than lack of variant pathogenicity. |
| 13 | Clinical Characterization and Mitochondrial DNA Sequence Variations in Leber Hereditary Optic Neuropathy | m.15287T>C | 23170061 | Pathogenicity was based predominantly on in silico prediction and structural modeling in a small mtDNA sequencing cohort, without segregation or direct variant-specific functional confirmation. |
| 14 | Novel m.15434C>A (p.230L>I) Mitochondrial Cytb Gene Missense Mutation Associated With Dilated Cardiomyopathy | m.15434C>A | 22811935 | Reported in a single patient, with pathogenicity supported mainly by absence from controls, conservation, and computational evidence; no segregation or direct functional confirmation was available, and the authors called for further experimental validation. |
| 15 | MT-CYB Mutations in Hypertrophic Cardiomyopathy | m.15024G>A; m.15482T>C | 24498601 | Both variants were described as putative/leaky disease-associated variants, with conclusions relying mainly on modeling; their primary causal role remained unresolved and required further study. |
| 16 | Association of the Level of Heteroplasmy of the 15059G>A Mutation in the MT-CYB Mitochondrial Gene With Essential Hypertension | m.15059G>A | 23710300 | Population association study rather than an individual MT-CYB-related mitochondrial disease report. The study evaluated blood heteroplasmy as a risk factor for essential hypertension and did not establish hypertension as a primary MT-CYB mitochondrial phenotype. |
| 17 | Mitochondrial DNA Variant m.15218A>G in Finnish Epilepsy Patients Who Have Maternal Relatives With Epilepsy, Sensorineural Hearing Impairment or Diabetes Mellitus | m.15218A>G | 23870133 | The variant is a common haplogroup U5a1-associated variant, was also present in population controls, and was characterized by the authors only as mildly deleterious rather than an established primary pathogenic mutation. |
| 18 | Private Mitochondrial DNA Variants in Danish Patients With Hypertrophic Cardiomyopathy | m.15024G>A | 25923817 | The authors’ subsequent detailed analysis explicitly concluded that the variant was unlikely to cause hypertrophic cardiomyopathy. |
| 19 | A Novel Mutation in the Mitochondrial DNA Cytochrome b Gene (MTCYB) in a Patient With Prader Willi Syndrome | m.15209T>C | 24771578 | The patient had a confirmed Prader–Willi syndrome diagnosis, providing an alternative genetic explanation for much of the phenotype; the homoplasmic MT-CYB variant lacked maternal segregation and direct functional confirmation, with pathogenicity largely based on conservation/in silico prediction. |
| 20 | Analysis of Mitochondrial Gene Mutations in a Child With Leigh Syndrome | (*MT-ND6, MTCYB*)-390 nt deletion | 30950016 | The patient carried the well-established pathogenic MT-ND3 m.10191T>C variant; the MT-ND6/MT-CYB deletion was also detected in the unaffected mother and brother. Thus, an independent causal contribution of the MT-CYB deletion could not be established. |
| 21 | Primary Hypoparathyroidism and Multiple Neuraxial Involvement in Mitochondrial Disorder Due to the Variant m.15043G>A in MT-CYB | m.15043G>A | 32334272 | Insufficient direct evidence of pathogenicity: no muscle biopsy, segregation analysis, or variant-specific functional study was provided; several manifestations could also be secondary to severe hypoparathyroidism/hypocalcemia. |
| 22 | Co-occurrence of m.15992A>G and m.15077G>A Is Associated With a High Penetrance of Maternally Inherited Hypertension in a Chinese Pedigree | m.15077G>A | 34427578 | The authors identified MT-TP m.15992A>G as the potentially pathogenic primary variant and proposed m.15077G>A as a secondary synergistic variant, rather than an independently pathogenic MT-CYB mutation. |
| 23 | Effective Treatment of Choreaballism Due to an MT-CYB Variant With Haloperidol, Tetrabenazine, and Antioxidants | m.15043G>A | 37351357 | The pathogenicity of m.15043G>A remains uncertain. The authors explicitly questioned its causal role, and previous studies have reported the variant in diverse clinical and genetic contexts, including classification as a nonpathogenic polymorphism. |
| 24 | Clinical and Genetic Analysis of Essential Hypertension With CYB Gene m.15024G>A Mutation | m.15024G>A | 37643984 | The study supports mitochondrial functional effects but concludes that the variant may play a synergistic role in essential hypertension; incomplete penetrance and prior evidence arguing against primary causality do not establish it as a primary pathogenic MT-CYB disease variant. |
| 25 | Investigating the Dual Role of Mitochondrial and Nuclear Genome Variants in Pediatric Cardiomyopathies | m.15734G>A | 40369053 | The patient carried multiple nuclear variants (including TNNT2, PLEC, and LAMA2) and another mtDNA variant, while the MT-CYB variant had only a moderate in silico pathogenicity score; the study emphasizes possible multivariant/synergistic effects rather than isolated MT-CYB causality. |

**Abbreviations:** LHON, Leber hereditary optic neuropathy; mtDNA, mitochondrial DNA; *MT-CYB*, mitochondrially encoded cytochrome b; *MT-CO3*, mitochondrially encoded cytochrome c oxidase III; *MT-ND3/MT-ND6*, mitochondrially encoded NADH dehydrogenase subunit 3/6.

**Table S4** Contemporary ClinVar and ClinGen classifications of *MT-CYB* variants included in this study

| **No.** | **Variant** | **Protein change** | **Current ClinVar classification** | **ClinVar review status** | **ClinGen Mitochondrial Diseases VCEP** | **ClinGen criteria / approval date** |
| --- | --- | --- | --- | --- | --- | --- |
| 1 | m.15615G>A | p.G290D | VUS | ★★★ Expert panel | VUS | PM2_Supporting, PP3; 2025-02-04 |
| 2 | m.15762G>A | p.G339E | No ClinVar variant-level record found | — | Not curated | — |
| 3 | m.15059G>A | p.G105* | VUS | ★ Single submitter | Not curated | — |
| 4 | m.15243G>A | p.G166E | VUS | ★ Single submitter | Not curated | — |
| 5 | m.14787_14790del | p.I14fs*50 | Likely pathogenic | ★★★ Expert panel | Likely pathogenic | PVS1_Strong, PM2_Supporting, PM6_Supporting; 2023-12-11 |
| 6 | m.15498_15521del | p.G251_L258del | No ClinVar variant-level record found | — | Not curated | — |
| 7 | m.14846G>A | p.G34S | VUS | ★★★ Expert panel | VUS | PS3_Supporting, PM2_Supporting, PP3; 2024-01-08 |
| 8 | m.15168G>A | p.W141* | No ClinVar variant-level record found | — | Not curated | — |
| 9 | m.15084G>A | p.W113* | No ClinVar variant-level record found | — | Not curated | — |
| 10 | m.15723G>A | p.W326* | No ClinVar variant-level record found | — | Not curated | — |
| 11 | m.15498G>A | p.G251D | VUS | ★ Single submitter | Not curated | — |
| 12 | m.15242G>A | p.G166* | Likely pathogenic | ★★★ Expert panel | Likely pathogenic | PVS1_Strong, PM2_Supporting, PS3_Supporting; 2024-01-08 |
| 13 | m.15579A>G | p.Y278C | VUS | ★★★ Expert panel | VUS | PS3_Supporting, PM2_Supporting, PP3; 2022-07-11 |
| 14 | m.15150G>A | p.W135* | Likely pathogenic | ★★★ Expert panel | Likely pathogenic | PVS1_Strong, PM2_Supporting; 2024-01-08 |
| 15 | m.15197T>C | p.S151P | VUS | ★★★ Expert panel | VUS | PM2_Supporting, PP3; 2024-01-08 |
| 16 | m.14849T>C | p.S35P | VUS | ★★★ Expert panel | VUS | PM2_Supporting, PP3; 2022-06-30**^†^** |
| 17 | m.15800C>T | p.Q352* | No ClinVar variant-level record found | — | Not curated | — |
| 18 | m.15761G>A | p.G339* | No ClinVar variant-level record found | — | Not curated | — |
| 19 | m.15170G>A | p.G142* | VUS | ★ Single submitter | Not curated | — |
| 20 | m.15699G>C | p.R318P | No ClinVar variant-level record found | — | Not curated | — |
| 21 | m.15635T>C | p.S297P | No ClinVar variant-level record found | — | Not curated | — |
| 22 | m.14849T>C | p.S35P | VUS | ★★★ Expert panel | VUS | PM2_Supporting, PP3; 2022-06-30**^†^** |
| 23 | m.14864T>C | p.C40R | No classification provided | 0 star | Not curated | — |
| 24 | m.15092G>A | p.G116S | No ClinVar variant-level record found | — | Not curated | — |
| 25 | m.15649_15666del | p.I302_P307del | No ClinVar variant-level record found | — | Not curated | — |
| 26 | m.15319_15327del | p.L192_T194del | No ClinVar variant-level record found | — | Not curated | — |
| 27 | m.15385_15405del | p.H215_F221del | No ClinVar variant-level record found | — | Not curated | — |
| 28 | m.15579A>G | p.Y278C | VUS | ★★★ Expert panel | VUS | PS3_Supporting, PM2_Supporting, PP3; 2022-07-11 |
| 29 | m.15045G>A | p.R100Q | VUS | ★ Single submitter | Not curated | — |
| 30 | m.15108T>G | p.L121R | No ClinVar variant-level record found | — | Not curated | — |
| 31 | m.14757T>C | p.M4T | VUS | ClinVar submission | Not curated | — |
| 32 | m.15309T>C | p.I188T | VUS | ★ Single submitter | Not curated | — |
| 33 | m.15635T>C | p.S297P | No ClinVar variant-level record found | — | Not curated | — |
| 34 | m.15045G>A | p.R100Q | VUS | ★ Single submitter | Not curated | — |

**Notes:** ClinVar and ClinGen classifications were accessed on September 3, 2026. ClinGen criteria and approval dates are reported only when a variant-level classification by the ClinGen Mitochondrial Diseases Variant Curation Expert Panel (VCEP) was confirmed. Historical or condition-specific ClinVar assertions may differ from the current aggregate classification. “Not curated” indicates that no variant-level ClinGen Mitochondrial Diseases VCEP classification was identified.

**†** For m.14849T>C, the Evidence Repository record displays an approval metadata date of 2022-06-30, while the narrative states that the VCEP classification was approved on 2022-05-24.

**Table S5** Mitochondrial respiratory chain enzyme activities in the concurrently assayed healthy muscle control and the patient

| **Sample / expression** | **CI** | **CII** | **CII+III** | **CIII** | **CIV** | **CS** |
| --- | --- | --- | --- | --- | --- | --- |
| Concurrent healthy control: % of normal | 20.80 | 22.80 | 70.40 | 20.60 | 91.40 | 65.80 |
| Concurrent healthy control: normalized to CS (%) | 31.60 | 35.30 | 105.60 | 32.10 | 130.70 | — |
| Concurrent healthy control: normalized to CII (%) | 87.30 | — | 290.70 | 89.10 | 350.00 | — |
| Patient: % of normal | 91.40 | 104.60 | 104.70 | 8.20 | 183.80 | 112.40 |
| Patient: normalized to CS (%) | 81.60 | 94.70 | 92.00 | 7.40 | 154.10 | — |
| Patient: normalized to CII (%) | 83.80 | — | 94.40 | 7.70 | 153.70 | — |

**Notes:** A skeletal muscle (biceps brachii) control from a healthy 15-year-old female was assayed concurrently with the patient sample as an internal assay control.

**Abbreviations:** CI–CIV, mitochondrial respiratory chain complexes I–IV; CS, citrate synthase.

**Table S6** RNA-seq expression and splicing profiles of key nuclear-encoded complex III-related genes

| **Gene** | **Category** | **TPM** | **Expression Z-score** | **Fold change** | **Expression *p* value** | **Adjusted *p* value** | **Significant FRASER splicing outlier** |
| --- | --- | --- | --- | --- | --- | --- | --- |
| UQCRC1 | Structural subunit | 106.53 | -0.69 | 0.95 | 0.477 | 1.00 | No |
| UQCRC2 | Structural subunit | 24.84 | -1.98 | 0.89 | 0.043 | 1.00 | No |
| CYC1 | Structural subunit | 204.32 | -2.04 | 0.87 | 0.040 | 1.00 | No |
| UQCRFS1 | Structural subunit | 62.25 | -1.18 | 0.92 | 0.226 | 1.00 | No |
| UQCRH | Structural subunit | 43.28 | 0.63 | 1.04 | 0.535 | 1.00 | No |
| UQCRB | Structural subunit | 43.21 | 0.38 | 1.03 | 0.718 | 1.00 | No |
| UQCRQ | Structural subunit | 143.10 | -0.06 | 0.99 | 0.937 | 1.00 | No |
| UQCR10 | Structural subunit | 88.79 | 0.27 | 1.02 | 0.804 | 1.00 | No |
| UQCR11 | Structural subunit | 48.89 | 0.05 | 1.00 | 0.975 | 1.00 | No |
| BCS1L | Assembly/maintenance | 41.36 | 0.16 | 1.01 | 0.897 | 1.00 | No |
| TTC19 | Assembly/maintenance | 9.81 | 1.86 | 1.16 | 0.051 | 1.00 | No |
| LYRM7 | Assembly/maintenance | 2.34 | 1.06 | 1.13 | 0.229 | 1.00 | No |
| UQCC1 | Assembly/maintenance | 3.42 | 0.44 | 1.04 | 0.673 | 1.00 | No |
| UQCC2 | Assembly/maintenance | 10.94 | -0.22 | 0.97 | 0.799 | 1.00 | No |
| UQCC3 | Assembly/maintenance | 31.34 | -1.11 | 0.91 | 0.268 | 1.00 | No |

**Notes:** Expression outliers were assessed using OUTRIDER. None of the 15 genes showed a significant expression outlier after multiple-testing correction (adjusted P = 1.00 for all genes). Splicing outliers were assessed using FRASER; no significant FRASER splicing outlier was identified in the selected complex III-related genes after multiple-testing correction. TPM, transcripts per million.

**Table S7** Summary of tissue-specific heteroplasmy data available in patients with *MT-CYB*-associated mitochondrial disease

| **Tissue sampling characteristics** | **MM (n=17)** | **Non-MM (n=16)** | **Total (n=33)** |
| --- | --- | --- | --- |
| **Number of tissues assessed** |  |  |  |
| 1 tissue | 2 (11.8%) | 1 (6.3%) | 3 (9.1%) |
| 2 tissues | 5 (29.4%) | 4 (25.0%) | 9 (27.3%) |
| 3 tissues | 9 (52.9%) | 4 (25.0%) | 13 (39.4%) |
| 4 tissues | 0 (0%) | 6 (37.5%) | 6 (18.2%) |
| 5 tissues | 1 (5.9%) | 1 (6.3%) | 2 (6.1%) |
| ≥ 2 tissues | 15 (88.2%) | 15 (93.8%) | 30 (90.9%) |
| **Tissues assessed** |  |  |  |
| Skeletal muscle | 17 (100%) | 13 (81.3%) | 30 (90.9%) |
| Blood | 15 (88.2%) | 14 (87.5%) | 29 (87.9%) |
| Fibroblast | 6 (35.3%) | 7 (43.8%) | 13 (39.4%) |
| Urine | 3 (17.6%) | 6 (37.5%) | 9 (27.3%) |
| Hair | 1 (5.9%) | 3 (18.8%) | 4 (12.1%) |
| Oral mucosa | 2 (11.8%) | 1 (6.3%) | 3 (9.1%) |
| Liver | 0 (0%) | 3 (18.8%) | 3 (9.1%) |
| Other tissuesᵃ | 0 (0%) | 3 (18.8%) | 3 (9.1%) |

**Notes:** Data are presented as n (%). The number of tissues assessed refers to the number of tissues for which tissue-specific heteroplasmy data were available. Case 31 was excluded because tissue-specific heteroplasmy data were unavailable. ᵃ Other tissues include heart, kidney, and ovary. MM, mitochondrial myopathy.
